# BAP1 promotes osteoclast function by metabolic reprogramming

**DOI:** 10.1038/s41467-023-41629-4

**Published:** 2023-09-22

**Authors:** Nidhi Rohatgi, Wei Zou, Yongjia Li, Kevin Cho, Patrick L. Collins, Eric Tycksen, Gaurav Pandey, Carl J. DeSelm, Gary J. Patti, Anwesha Dey, Steven L. Teitelbaum

**Affiliations:** 1 Division of Anatomic and Molecular Pathology, Department of Pathology and Immunology, Washington University School of Medicine, St. Louis, MO 63110 USA; 2 Department of Pharmacology, Jiangsu University School of Medicine, Zhenjiang, Jiangsu Province 212013 PR China; 3 Department of Chemistry, Washington University in St. Louis, St. Louis, MO 63130 USA; 4 Department of Medicine, Washington University School of Medicine, St. Louis, MO 63110 USA; 5 Center for Metabolomics and Isotope Tracing, Washington University in St. Louis, St. Louis, MO 63130 USA; 6 Department of Microbial Infection and Immunity, Ohio State University, Columbus, OH 43210 USA; 7 Genome Technology Access Center, McDonnell Genome Institute, Washington University School of Medicine, St Louis, MO 63110 USA; 8 Department of Radiation Oncology, Washington University School of Medicine, St. Louis, MO 63110 USA; 9 Department of Discovery Oncology, Genentech Inc., South San Francisco, CA 94080 USA; 10 Division of Bone and Mineral Diseases, Department of Medicine, Washington University School of Medicine, St. Louis, MO 63110 USA

**Keywords:** Post-translational modifications, Cytoskeleton, Bone, Energy metabolism

## Abstract

Treatment of osteoporosis commonly diminishes osteoclast number which suppresses bone formation thus compromising fracture prevention. Bone formation is not suppressed, however, when bone degradation is reduced by retarding osteoclast functional resorptive capacity, rather than differentiation. We find deletion of deubiquitinase, *BRCA1-associated protein 1 (Bap1)*, in myeloid cells (*Bap1*^*∆LysM*^), arrests osteoclast function but not formation. *Bap1*^*∆LysM*^ osteoclasts fail to organize their cytoskeleton which is essential for bone degradation consequently increasing bone mass in both male and female mice. The deubiquitinase activity of BAP1 modifies osteoclast function by metabolic reprogramming. *Bap1* deficient osteoclast upregulate the cystine transporter, *Slc7a11*, by enhanced H2Aub occupancy of its promoter. SLC7A11 controls cellular reactive oxygen species levels and redirects the mitochondrial metabolites away from the tricarboxylic acid cycle, both being necessary for osteoclast function. Thus, in osteoclasts BAP1 appears to regulate the epigenetic-metabolic axis and is a potential target to reduce bone degradation while maintaining osteogenesis in osteoporotic patients.

## Abstract (web summary)

Here, the authors demonstrate that BRCA1-associated protein 1 (Bap1) regulates osteoclast’s capacity to degrade bone. Reprogramming of epigenetic-metabolic axis upon Bap1 loss inhibits bone degradation, preserving bone mass, making it a potential therapeutic target for osteoporosis.

## Introduction

Bone remodeling, which maintains skeletal health, requires an equilibrium of osteogenic osteoblasts and resorptive osteoclasts. When this coupling is deranged, the balance between formation and resorption is lost, resulting in osteoporosis which is endemic in Western society. Osteoclasts are polykaryons derived from bone-marrow macrophages (BMMs) exposed to receptor activators of nuclear factor kappa-Β ligand (RANKL) and macrophage colony-stimulating factor (M-CSF)^1^. Net bone resorption is the product of both osteoclast abundance and the matrix-degrading capacity of the individual polykaryon. These processes differ in regard to the treatment of osteoporosis. While therapeutically diminished osteoclast number also suppresses bone formation, osteoblast activity is spared when an abundance of the resorptive cell is maintained, but only its degradative capacity is inhibited. During the process of resorption, osteoclasts adhere to the bone surface and form a “gasket-like” structure, known as the actin ring or sealing zone, to isolate the resorptive microenvironment from the general extracellular space^2,3^. The signals that govern cytoskeleton organization to form actin rings therefore dictate the cell’s capacity to resorb bone and impact skeletal integrity.

While osteoclasts are generated and function via genetic and post-transcriptional regulation, epigenetic events are equally important to the process. Our previous studies highlight that an epigenetic factor, ASXL2, is necessary for optimal osteoclast formation, and its absence in myeloid lineage cells substantially increases bone mass due to a paucity of osteoclasts^4,5^. ASXL2 binds an enzymatic protein, BAP1, to form a deubiquitinase (PR-DUB) complex that removes monoubiquitin from substrates such as histone 2A^6,7^.

Given that ASXL2 and BAP1 physically interact, we postulated that BAP1 would similarly modulate the osteoclast. In keeping with this hypothesis, we find that *Bap1* deletion in myeloid lineage cells, like that of *Asxl2*, dampens bone resorption, thereby increasing skeletal mass. Unexpectedly, unlike ASXL2, which suppresses bone resorption by arresting osteoclast differentiation, myeloid absence of BAP1 enhances bone mass by metabolically impairing cytoskeletal organization. In addition to altered amino acid metabolism, BAP1 targets the cystine transporter, SLC7A11, which modulates antioxidant glutathione abundance and mitochondrial flux in osteoclasts. The fact that BAP1 arrests osteoclast function epigenetically raises the possibility that its therapeutic potential may be superior to present anti-resorptive drugs.

## Results

### *Bap1*^*∆LysM*^ mice are osteopetrotic

We identified the epigenetic factor ASXL2 as a positive regulator of osteoclast formation^4^. Because of its known association with a deubiquitinase, BAP1, to maintain protein stability^8,9^, we asked if *Bap1* deficiency, like that of *Asxl2*, increases bone mass by arresting osteoclast differentiation, thereby diminishing the abundance of the bone resorptive cell.

**Fig. 1:**
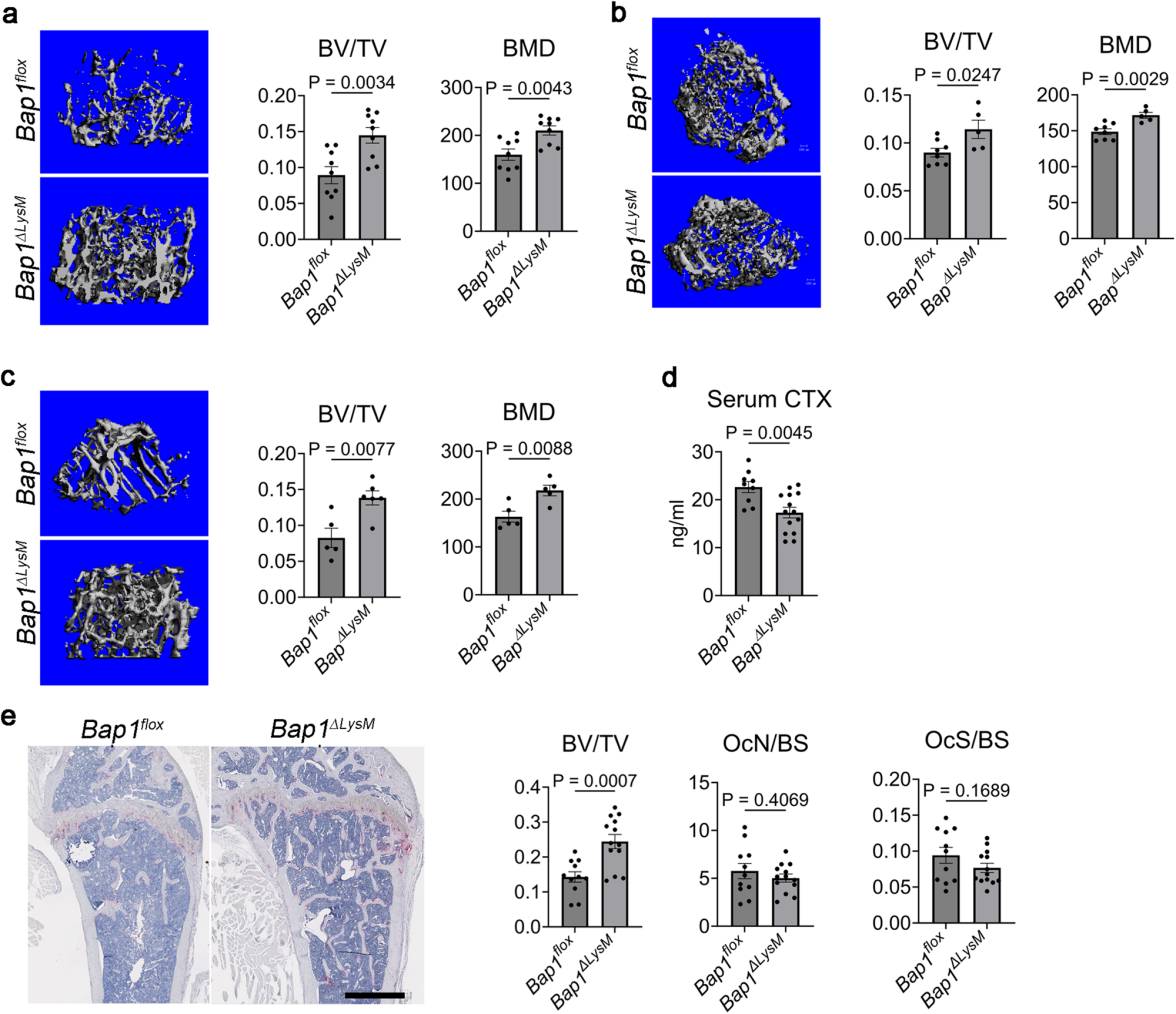
*Bap1*^*∆LysM*^ mice are osteopetrotic. **a**, **b** µCT images and trabecular bone parameters of 20-week-old *Bap1*^*flox*^ and *Bap1*^*∆LysM*^ **a** male; *n* = 9 biologically independent samples and **b** female; *n* = 8 (*Bap1*^*flox*^) and *n* = 5 (*Bap1*^*∆LysM*^) biologically independent samples. **c** µCT images and trabecular bone parameters of 1-year-old *Bap1*^*flox*^ and *Bap1*^*∆LysM*^ male littermates; *n* = 5 biologically independent samples. **d** Serum CTX of 20-week-old male *Bap1*^*flox*^ and *Bap1*^*∆LysM*^ mice; *n* = 9 (*Bap1*^*flox*^) and *n* = 14 (*Bap1*^*∆LysM*^) biologically independent samples. **e** (Left) Representative femur of a 20-week-old male *Bap1*^*flox*^ and *Bap1*^*∆LysM*^ mice stained for TRAP activity (red reaction product). (Right) Histomorphometric analysis of trabecular bone; *n* = 11 (*Bap1*^*flox*^*)* and *n* = 13 (*Bap1*^*∆LysM*^) biologically independent samples. The scale bar represents 1 mm. OcN osteoclast number, OcS osteoclast surface, BS bone surface, BV trabecular bone volume, TV total volume, BV/TV bone volume fraction of marrow, vBMD volumetric bone mineral density. Data represent mean ± SEM. *P* values shown are by two-sided Student’s *t*-test (**a**–**e**). Source data are provided as a Source Data file.

To determine the role of BAP1 in osteoclasts, we generated mice in which the gene is exclusively deleted in myeloid lineage cells (*Bap1*^***∆****LysM*^) (Supplementary Fig. 1a). At 20 weeks of age, femoral trabecular bone mass (BV/TV) and bone mineral density (BMD) are increased in *Bap1*^***∆****LysM*^ male and female mice (Fig. 1a, b). Trabecular connectivity, spacing, and number are also significantly changed in keeping with enhanced bone mass (Supplementary Fig. 1b, c). Myeloid deletion of the gene is similarly effective in 1-year-old mice (Fig. 1c and Supplementary Fig. 1d). Establishing the increased bone mass reflects decreased resorption, serum CTx (c-terminal telopeptides type I collagen), a marker of bone matrix degradation, in vivo, is significantly reduced in *Bap1*^*∆LysM*^ mice (Fig. 1d). Interestingly, while histomorphometric analysis also reflect increased trabecular bone mass (BV/TV) in the *Bap1*^*∆LysM*^ mice, no significant changes were noted in either osteoclast number or osteoclast surface (Fig. 1e).

### BAP1 regulates osteoclast function

**Fig. 2:**
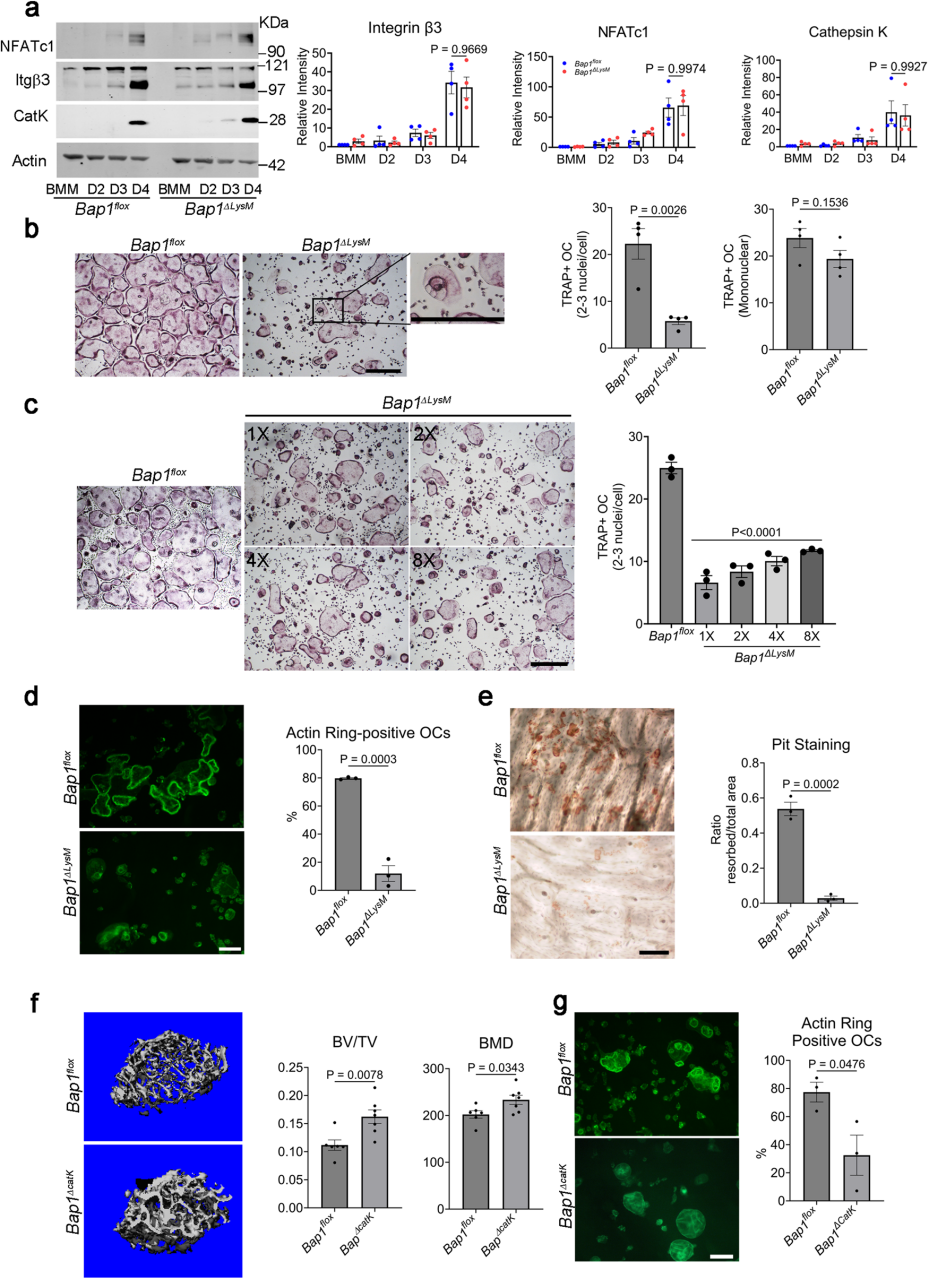
BAP1 regulates osteoclast function. **a** A 8-week-old *Bap1*^*flox*^ and *Bap1*^*∆LysM*^ BMMs were cultured with M-CSF and RANKL (100 ng/mL). The total cell lysate was collected with time. (Left) Representative immunoblot for the osteoclast differentiation proteins. (Right) Densitometric analysis of osteoclast differentiation proteins; *n* = 4 independent experiments. **b** (Left) Representative image of *Bap1*^*flox*^ and *Bap1*^*∆LysM*^ osteoclasts stained for TRAP activity. The scale bar represents 500 µm. Inset shows TRAP-positive *Bap1*^*∆LysM*^ osteoclast at a higher magnification; *n* = 4 independent experiments. (Right) Quantification of multinuclear and mononuclear TRAP +ve cells in either genotype. **c** Two- to eightfold more *Bap1*^*∆LysM*^ BMMs in contrast to *Bap1*^*flox*^ BMMs (1×) were cultured with M-CSF and RANKL (100 ng/mL) for 5 days to form osteoclasts (OC), after which they were stained for TRAP activity. The scale bar represents 500 µm; *n* = 3 independent experiments. **d** *Bap1*^*flox*^ and *Bap1*^*∆LysM*^ BMMs were cultured with M-CSF and RANKL (100 ng/mL) on bovine bone slices. After 5 days, the cells were stained with Alexa-Fluor-488-Phallodin to visualize the actin rings (green color) which were quantified (right panel); Scale bar represents 100 μm; *n* = 3 independent experiments. **e** Resorption pits were visualized by wheat germ agglutinin–lectin staining, and the resorbed surface was quantified (right panel). The scale bar represents 100 µm; *n* = 3 independent experiments. **f** µCT analysis on femurs of 20-week-old male *Bap1*^*flox*^ and *Bap1*^*∆catK*^ littermates; *Bap1*^*flox*^ *n* = 6 and *Bap1*^*∆catK*^ *n* = 7 biologically independent samples. BV/TV bone volume fraction of marrow, vBMD volumetric bone mineral density analyzed. **g** Actin rings for *Bap1*^*flox*^ and *Bap1*^*∆catK*^ osteoclasts cultured on bone slices. The % of osteoclasts exhibiting actin rings was quantified. The scale bar represents 100 µm; *n* = 3 independent experiments. Data represent mean ± SEM. *P* values shown are by two-way ANOVA with Sidak multiple comparison testing (**a**) or two-sided Student’s *t*-test (**b**, **d**–**g**) or one-way-ANOVA with Dunnett’s testing (**c**). Source data are provided as a Source Data file.

The substantially enhanced bone mass and decreased resorption in *Bap1*^*∆LysM*^ mice suggests that *Bap1* deficiency may impact osteoclast formation, which is contradicted, however, by the normal abundance of the bone resorptive cell (Fig. 1e). To explore this conundrum, we cultured *Bap1*^*∆LysM*^ bone marrow macrophages (BMMs) with the key osteoclastogenic cytokines, M-CSF and RANKL. The osteoclast differentiation markers NFATc1, β3 integrin, and Cathepsin K are unaltered in those lacking BAP1 (Fig. 2a), establishing the increased bone mass of *Bap1*^***∆****LysM*^ mice does not reflect arrested osteoclast differentiation. Normal bone formation, as determined by dynamic histomorphometry (Supplementary Fig. 2a) and unaltered osteoclast differentiation in *Bap1*^***∆****LysM*^ mice, suggests their enhanced skeletal mass may represent the diminished resorptive capacity of individual osteoclast. Indeed, BMM-derived *Bap1*^***∆****LysM*^ TRAP-positive cells mirror those which are β3 integrin deficient, as they abnormally spread and appear “crenated”^10^ (Fig. 2b). While the number of large, multinucleated TRAP expressing cells are decreased in RANKL/M-CSF stimulated cultures of *Bap1* deficient BMMs, no significant difference exists between the abundance of all TRAP+ cells consistent with failure to normally spread. Alamar Blue assay that measures cell number confirms that *Bap1*^***∆****LysM*^ and *Bap1*^*flox*^ BMMs are equally proliferative (Supplementary Fig. 2b).

In normal circumstances, increasing the number of osteoclast precursors in culture facilitates their capacity to spread, fuse, and form characteristic multinucleated cells. In contrast, enhancing the abundance of *Bap1*^***∆****LysM*^ BMMs does not alter the crenated appearance of *Bap1* deficient osteoclasts or increase their number, establishing this phenotype does not reflect precursor abundance (Fig. 2c). To understand the functional consequence of the abnormal appearance of *Bap1*^***∆****LysM*^ osteoclasts, we assessed their bone resorptive capacity. Equal numbers of *Bap1*^*flox*^ or *Bap1*^***∆****LysM*^ BMMs were cultured on bone slices. 60% of *Bap1*^*flox*^ osteoclasts form detectable actin rings which encompass the resorptive microenvironment and are essential for bone degradation. In contrast and confirming cytoskeletal dysfunction, actin rings are essentially absent in *Bap1*^***∆****LysM*^ osteoclasts (Fig. 2d). *Bap1*^*flox*^ osteoclasts resorb approximately 50% of the bone surface while *Bap1*^***∆****LysM*^ cultures generate no resorption lacunae (Fig. 2e). Thus, absence of Bap1 in myeloid lineage cells arrests osteoclast function. Such being the case, we asked if *Bap1* elimination only in mature osteoclasts similarly induces osteopetrosis. In fact, Bap1 deletion, using cathepsin K-cre, which targets cells committed to the osteoclast phenotype, increases bone mass (Fig. 2f and Supplementary Fig. 2c, d) and eliminates actin rings (Fig. 2g). Hence, myeloid deficiency of *Bap1* inhibits bone resorption by dampening resorptive capacity of mature osteoclasts.

### BAP1 epigenetic activity modulates osteoclast function

**Fig. 3:**
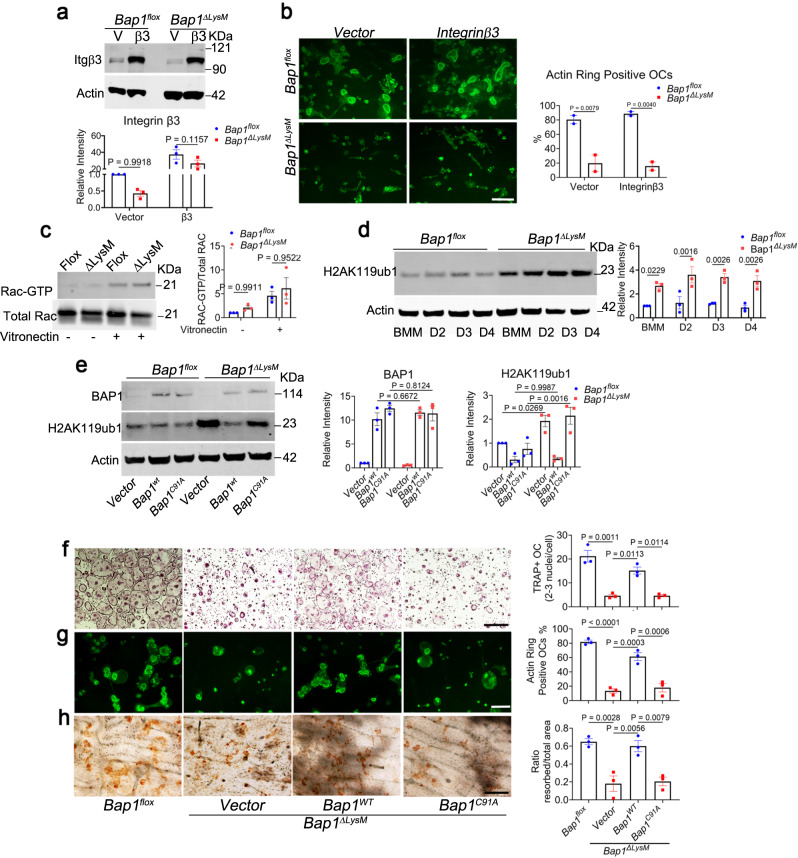
BAP1 epigenetic activity modulates osteoclast function. **a** Representative immunoblot of integrin *β3* expression in *Bap1*^*flox*^ and *Bap1*^*∆LysM*^ osteoclast retrovirally transduced with *integrin β3* or *vector* (BMM from 8- to 12-week-old male mice); *n* = 3 biologically independent experiments. **b** Transduced BMMs were cultured with M-CSF and RANKL (100 ng/mL) on bovine bone slices. After 5 days, the cells were stained with Alexa-Fluor-488-Phallodin to visualize the actin rings (green color), and % of osteoclasts exhibiting actin rings per well were quantified. The scale bar represents 100 µm; *n* = 2 independent experiments. **c** Day 3 *Bap1*^*flox*^ and *Bap1*^*∆LysM*^ pre-osteoclasts (preOCs) from 8- to 12-week-old male mice were cultured for 3 h in serum-free medium. The cells were plated on a vitronectin-coated petri dish (+) or maintained in suspension for 30 min (−). GTP-Rac1 and total Rac1 were analyzed by pull-down assay followed by immunoblot, *n* = 3 independent experiments. **d** *Bap1*^*flox*^ *and Bap1*^*∆LysM*^ BMMs were cultured with M-CSF ± RANKL (100 ng/mL). The total cell lysate was collected with time. The abundance of H2AK119ub1 modification was determined by immunoblot; *n* = 3 biologically independent experiments. **e** *Bap1*^*∆LysM*^ BMMs from 16- to 20-week-old male mice transduced with human *Bap1*^*WT*^; *Bap1*^*C91A*^ or vector (V) were exposed to M-CSF (BMM) or M-CSF and RANKL (100 ng/mL) for 3 days to generate pre-osteoclasts (preOCs). The total cell lysate was extracted, and BAP1 or H2AK119ub1 expression was determined by immunoblot; *n* = 3 independent experiments. **f** Fully differentiated osteoclasts were stained for TRAP activity. The scale bar represents 500 µm; *n* = 3 independent experiments. **g** Cells stained with 488-Phalloidin to visualize actin rings (green color). % of osteoclasts exhibiting actin rings was quantified. The scale bar represents 100 µm; *n* = 3 independent experiments. **h** Following removal of the transduced osteoclasts, resorption pits were visualized by wheat germ agglutinin-lectin staining and quantified per well. The scale bar represents 100 μm; *n* = 3 independent experiments. Data are represented as mean ± SEM. *P* values shown are by two-way ANOVA with Sidak’s multiple comparison testing (**a**–**e**) or one-way ANOVA with Tukey’s post-hoc testing (**f**–**h**). Source data are provided as a Source Data file.

Defective individual osteoclast function is frequently a manifestation of failed cytoskeletal organization, which may be the product of the inactivation of the α_v_β_3_ integrin or its downstream effector molecules^10,11^. Their morphology suggested a possible alteration in cytoskeletal organization mediated by a paucity of α_v_β_3_ (Fig. 2b). To assess the possibility of compromised *integrin* β3 in *Bap1*^***∆****LysM*^ osteoclasts, we overexpressed *WT β3* in *flox* and *Bap1* deleted macrophages (Fig. 3a). In contrast to cells genetically lacking the integrin, transduced *β3* does not rescue the crenated appearance nor actin ring formation of *Bap1*^***∆****LysM*^ osteoclasts (Fig. 3b and Supplementary Fig. 3a). Since integrin-initiated osteoclast cytoskeletal organization is induced by a canonical pathway in which c-Src and RAC1 are critical components, we asked if *Bap1* deficiency disrupts these downstream effectors. To this end, we plated *Bap1*^*flox*^ or *Bap1*^***∆****LysM*^ pre-osteoclasts (preOC, defined as TRAP^+^ mononuclear and binuclear cells) on vitronectin which contains an RGD motif that activates α_v_β_3_ signaling. Neither phosphorylation of c-Src (Y416) (Supplementary Fig. 3b) nor GTP-associated RAC (Fig. 3c) differs from control in vitronectin-exposed *Bap1*^***∆****LysM*^ preOCs. Thus, the absence of BAP1 compromises the osteoclast cytoskeleton by a mechanism independent of the α_v_β_3_-induced signaling pathway.

BAP1functions as a deubiquitinase which removes ubiquitin moieties from outer mitochondrial membrane (OMM) proteins^12,13^. We find, however, BAP1 does not deubiquitinate OMM proteins in osteoclasts, as established by VDAC co-IP using ubiquitin and VDAC antibodies, even in the presence of the proteasomal inhibitor, MG132 (Supplementary Fig. 3c). Furthermore, incubation of preOCs with a fluorophore, TMRM dye followed by the depolarizing agent, FCCP yields a similar decrease in membrane potential in both groups (Supplementary Fig. 3d). We therefore turned to another established property of BAP1, namely modification of H2A. Indeed, immunoblot of H2AK119ub1 reveals increased bulk histone ubiquitination in *Bap1*^***∆****LysM*^ macrophages and differentiating osteoclasts (Fig. 3d). To determine if this enzymatic activity of BAP1 regulates osteoclast function, we overexpressed the human *WT* gene (*BAP1*^*WT*^*)* or its mutant (*BAP1*^*C91A*^) that lacks deubiquitinase activity, in *Bap1*^*flox*^ and *Bap1*^***∆****LysM*^ macrophages. Since commercially available BAP1 antibodies only recognize human BAP1, immunoblots for BAP1 show no endogenous mouse BAP1 protein in vector groups of either genotype (*Bap1*^*flox*^ vs. *Bap1*^***∆****LysM*^*)*. As expected, human BAP1 expression in osteoclasts transduced with both *BAP1* constructs was similar (Fig. 3e). Importantly, *BAP1*^*WT*^ but not *BAP1*^*C91A*^ reduces ubiquitination of histone H2A (Fig. 3e). Fortifying the concept that its enzymatic activity is required for osteoclast cytoskeleton organization, *WT* but not ubiquitinase-deficient *BAP1* normalizes the size of *Bap1*^***∆****LysM*^ osteoclasts (Fig. 3f and Supplementary Fig. 3e). Importantly, restoration of BAP1 deubiquitinase activity rescues actin ring formation and the cell’s resorptive capacity as evidenced by pit staining in *Bap1*^***∆****LysM*^ osteoclast cultures (Fig. 3g, h).

### BAP1 regulates osteoclast transcription

**Fig. 4:**
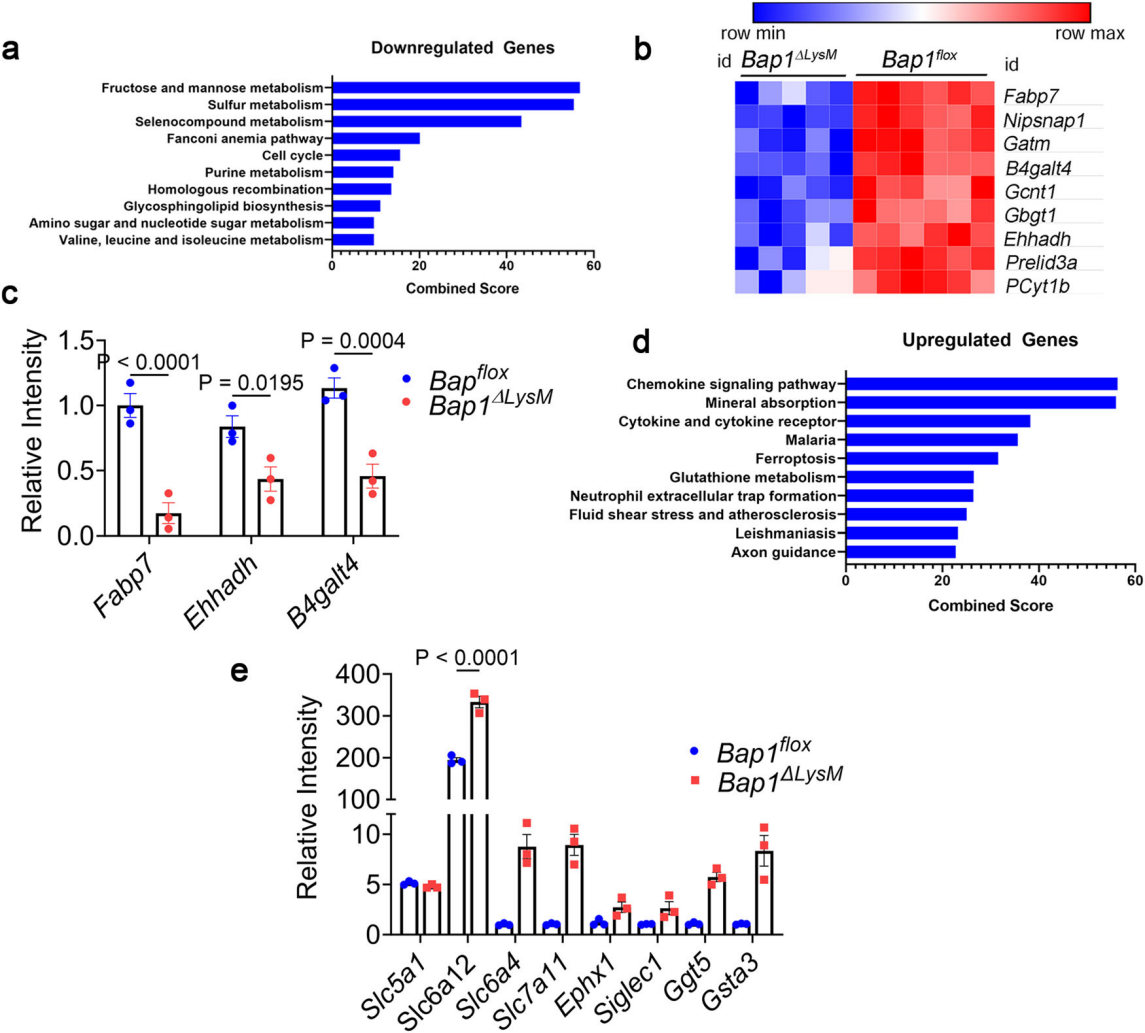
BAP1 regulates osteoclast transcription. RNA-seq analysis of osteoclasts derived from 20-week-old *Bap1*^*flox*^ and *Bap1*^*∆LysM*^ male mice. **a** Enrichr pathway analysis of all genes significantly downregulated (negative enrichment) in *Bap1*^*ΔLysM*^ preOCs. **b** Heatmap of most significantly downregulated genes in preOCs based on log (fold change) > 1.5 with adjusted *P* < 0.001 as determined by Benjamini–Hochberg false-discovery rate; *n* = 6 (*Bap1*^*flox*^) and *n* = 5 (*Bap1*^*∆LysM*^). **c** qPCR analysis of candidate genes involved in metabolism, *Fabp7, Ehhadh*, and *B4galt4* mRNA in *Bap1*^*flox*^ and *Bap1*^*∆LysM*^ preOCs; *n* = 3 independent experiments. **d** Enrichr pathway analysis of all genes significantly upregulated (positive enrichment, red bar) in *Bap1*^*flox*^ preOCs. **e** qPCR analysis of upregulated genes in *Bap1*^*flox*^ and *Bap1*^*∆LysM*^ BMMs and preOCs; *n* = 3 independent experiments. Data represents mean ± SEM. *P* values shown are by two-way ANOVA with Sidak’s multiple comparison testing (**c**, **e**). Source data are provided as a Source Data file.

To determine if histone deubiquitination by BAP1 regulates gene expression, we conducted unbiased genome-wide analyses to characterize transcriptional alterations related to *Bap1* deletion. *Bap1*^***∆****LysM*^ and control BMMs were treated with RANKL for 24 h to identify genetic changes that precede the observed differences in resorption and cytoskeletal organization. RNA sequencing (RNA-seq) analysis identifies 6075 differentially expressed genes (DEGs) (adj.p.val < 0.05). Of these DEGs, 499 are upregulated and 782 downregulated in *Bap1*^***∆****LysM*^ osteoclasts (fold change ≥ 1.5). PCA plot denotes separation by genotype (Supplementary Fig. 4a).The 50 genes undergoing the greatest change are represented in the heat map and volcano plot (Supplementary Fig. 4b, c). Pathway analysis reveals that downregulated DEGs include an abundance of those modulating metabolic pathways, particularly branched-chain amino acid metabolism (BCAA) (Fig. 4a). Furthermore, the most downregulated genes are associated with metabolism and mitochondrial function (Fig. 4b). Gene expression analysis of 3 candidate genes involved in metabolism validate RNAseq data and supports the concept of *Bap1* deletion on metabolic pathways in preOCs (Fig. 4c). In contrast, upregulated DEGs are enriched in glutathione metabolism and ferroptosis (Fig. 4d). Expression analysis of candidate genes validate upregulation of many solute carriers and glutathione pathway genes (Fig. 4e).

### BAP1 regulates *Slc7a11* expression by reducing the H2Aub occupancy of its promoter

**Fig. 5:**
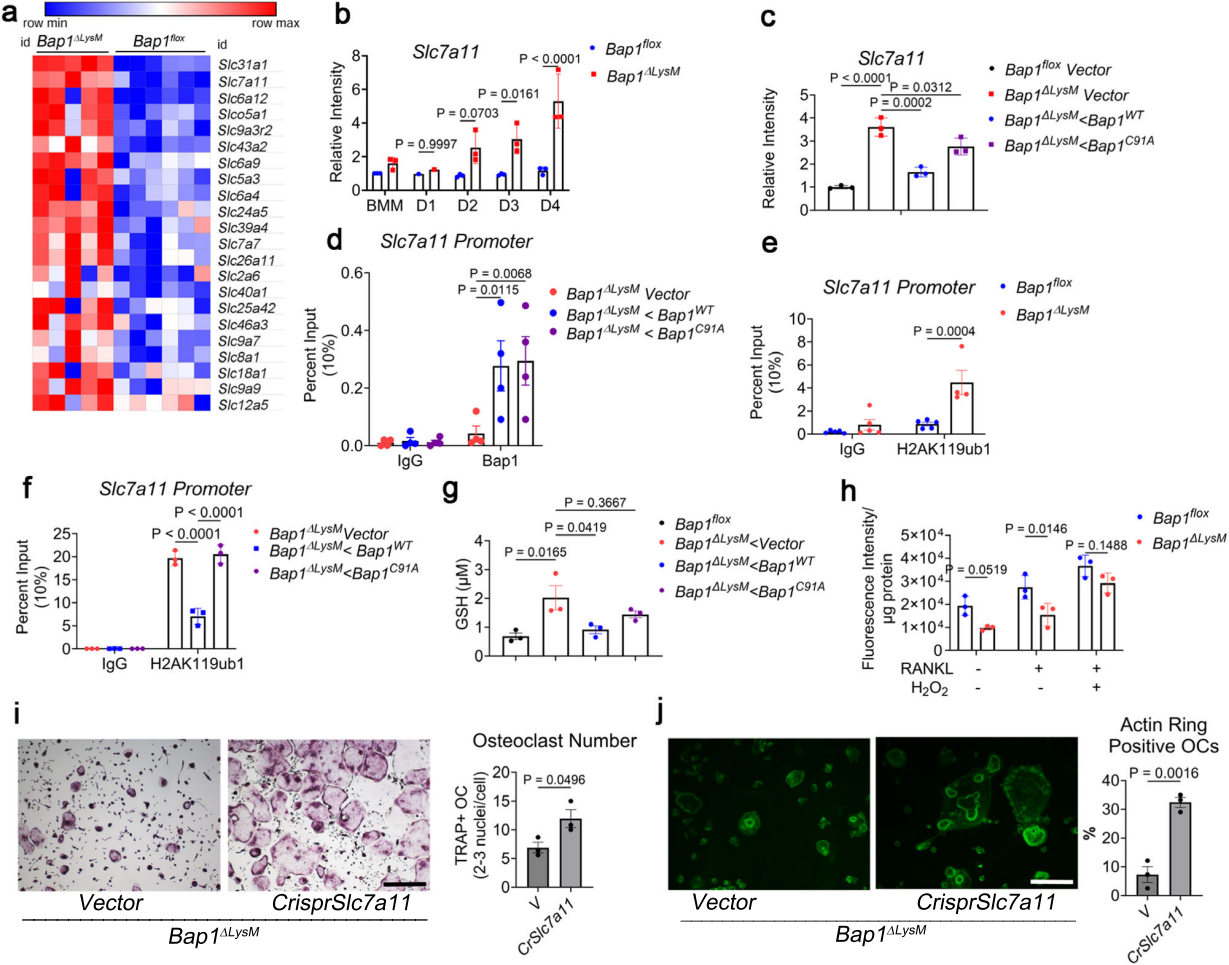
BAP1 regulates *Slc7a11* expression by reducing the H2Aub occupancy of its promoter. **a** Heatmap of solute carrier genes upregulated in preOCs based on log (fold change) > 1.5 with adjusted *P* < 0.001 as determined by Benjamini–Hochberg false-discovery rate; *n* = 6 (*Bap1*^*flox*^) and *n* = 5 (*Bap1*^*∆LysM*^) 20-week-old biologically independent samples. **b** qPCR analysis of *Slc7a11* mRNA in 16–20-week-old *Bap1*^*flox*^ *and Bap1*^*∆LysM*^ BMMs and osteoclasts at different stages of differentiation; *n* = 3 independent experiments. **c** *Bap1*^*∆LysM*^ BMMs, transduced with *BAP1*^*WT*^ or *BAP1*^*C91A*^ followed by treatment with M-CSF and RANKL (100 ng/mL) for 3 days. *Slc7a11* gene expression was determined by qPCR analysis; *n* = 3 independent experiments. **d** ChIP-qPCR quantifying BAP1 binding on *Slc7a11* promoter of *Bap1*^*∆LysM*^ preOCs transduced with either *BAP1*^*WT*^ or *Bap1*^*C91A*^ or *Vector*; *n* = 4 independent experiments. **e** ChIP-qPCR of H2Aub binding on the *Slc7a11* promoter in *Bap1*^*∆LysM*^ in comparison to the *Bap1*^*flox*^ preOCs *n* = 4 independent experiments. **f** ChIP-qPCR of H2Aub binding on the *Slc7a11* promoter in *Bap1*^*∆LysM*^ transduced with *Bap1*^*WT*^ or *Bap1*^*C91A*^ constructs; *n* = 3 independent experiments. **g** Representative graph of GSH abundance in *Bap1*^*∆LysM*^ preOCs transduced with *Vector*, *BAP1*^*wt*^, or *BAP1*^*C91A*^. *Bap1*^*flox*^ serves as control; *n* = 3 independent experiments. **h** ROS levels in *Bap1*^*flox*^ and *Bap1*^*ΔLysM*^ BMMs and OCs. Cells treated with H_2_O_2_ were used as a positive control. *n* = 3 independent experiments. **i**, **j** *Slc7a11* was knockdown using CRISPR in *Bap1*^*∆LysM*^ macrophages followed by exposure to MCSF and RANKL (100 ng/ml); *n* = 3 independent experiments. **i** Cells were stained for TRAP activity. Scale bar represents 500 µm, or **j** cultured on bone slices and stained with Alexa Flour-488-Phalloidin (green color) to visualize actin rings and quantified per well (right panel). The scale bar represents 100 µm. Data are represented as mean ± SEM. *P* values shown are by two-way ANOVA with Sidak’s multiple comparison testing (**b**, **d**–**f**, **h**) or one-way ANOVA with Tukey’s multiple comparison testing (**c**, **g**) two-sided Student**’**s *t-*test (**i**, **j**). Source data are provided as a Source Data file.

RNAseq analysis reveal upregulation of *Slc7a11* among other solute carrier proteins in *Bap1*^***∆****LysM*^ preOCs (Fig. 5a). SLC7A11 being an antiporter exchanges extracellular cystine, a major component of glutathione (GSH) biosynthesis, for intracellular glutamate^12^. It is also a direct target of BAP1 and regulates cellular GSH levels in renal cancer cells^12^. Importantly, qPCR analysis demonstrates increased *Slc7a11* mRNA expression in *Bap1*^***∆****LysM*^ in comparison to *Bap1*^*flox*^ preOCs (Fig. 5b). Additionally, its normalization by reintroduction of *BAP1*^*WT*^ but not *BAP1*^*C91A*^, suggests BAP1-mediated repression of *Slc7a11* expression requires BAP1’s DUB activity (Fig. 5c). To determine role of BAP1 in regulating *Slc7a11*, ChIP assay using BAP1 antibody was performed. Overexpressed *BAP1*^*WT*^ or *BAP1*^*C91A*^ binds directly to the *Slc7a11* promoter (Fig. 5d). In keeping with our hypothesis that BAP1 regulates *Slc7a11* expression epigenetically, H2AK119ub1 (H2AuB) antibody-based ChIP demonstrates increased H2Aub1 occupancy at various sites of the *Slc7a11* promoter in *Bap1* deficient preOCs (Fig. 5e and Supplementary Fig. 5a, b). Importantly, *BAP1*^*WT*^ but not *BAP1*^*C91A*^ decreases H2Aub1 occupancy of *Slc7a11* promoter (Fig. 5f). Thus, BAP1- repression of *Slc7a11* in osteoclasts requires BAP1’s DUB activity and H2Aub deubiquitination on the *Slc7a11* promoter.

To confirm if BAP1 regulates GSH biosynthesis by regulating the cystine transporter, we measured GSH in *Bap*^*flox*^ and *Bap1*^***∆****LysM*^ osteoclasts. GSH is, in fact, enhanced in *Bap1*^***∆****LysM*^ osteoclasts relative to their non-depleted counterpart (Fig. 5g). Furthermore, the increased GSH is diminished by the reintroduction of *BAP1*^*WT*^ but not mutant in *Bap1* deficient osteoclasts underscoring that regulation of GSH, in these cells, is mediated by BAP1 (Fig. 5g). Since GSH is the key cellular antioxidant, we assessed its effect on cellular redox level, especially because reactive oxygen species (ROS) are important for bone resorption^14–18^. We measured ROS in BMMs and preOCs and, consistent with impaired resorptive capacity, found it significantly reduced in *Bap1* deficiency (Fig. 5h).

To understand the influence of SLC7A11 on osteoclast in the absence of *Bap1*, we deleted the transporter by Crispr in *Bap1*^***∆****LysM*^ cells (Supplementary Fig. 5c). *Slc7a11* elimination substantially normalizes the crenated appearance of *Bap1*-deficient osteoclasts (Fig. 5i) and actin ring formation (Fig. 5j).

### BAP1 regulates mitochondrial respiration in osteoclasts

**Fig. 6:**
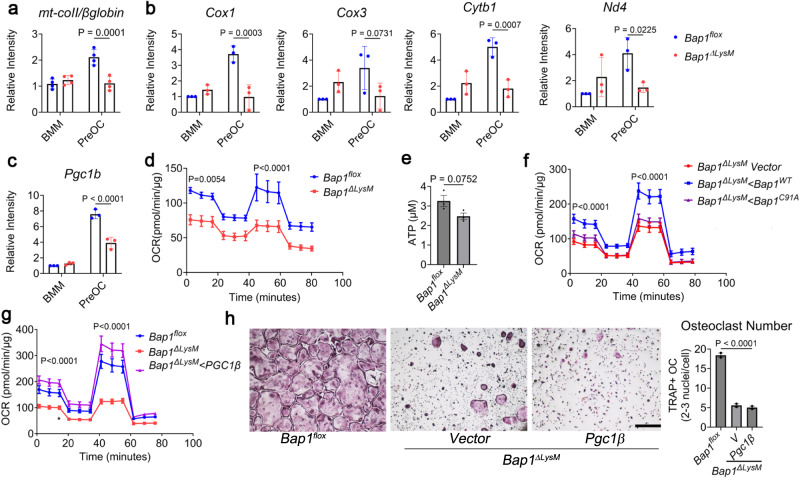
BAP1 regulates mitochondrial respiration in osteoclasts. *Bap1*^*flox*^ and *Bap1*^*∆LysM*^ BMMs derived from 16- to 20-week-old male mice were cultured with M-CSF ± RANKL for 5 days. **a** Relative abundance of mitochondria determined by qPCR of mt-Co2 DNA normalized to β-globin; *n* = 4 independent experiments. **b** Abundance of mitochondrial biogenesis marker mRNA determined by qPCR in macrophages (BMM) and preOCs; *n* = 3 independent experiments. **c** qPCR analysis of *Pgc1β* mRNA in *Bap1*^*flox*^ *and Bap1*^*∆LysM*^ BMMs and preOCs *n* = 3 independent experiments. **d** *Bap1*^*flox*^ *and Bap1*^*∆LysM*^ BMMs were cultured with M-CSF and RANKL for 3 days. Oxygen consumption rate (OCR) was analyzed by XF Cell Mito Stress Assay; *n* = 4 independent experiments. **e** Intracellular ATP abundance in preOCs derived from either genotype; *n* = 3 independent experiments. **f** Representative graph of OCR of *Bap1*^*∆LysM*^ BMMs, transduced with human *Bap1*^*WT*^; *Bap1*^*C91A*^ or *Vector (V)* exposed to M-CSF and RANKL (100 ng/mL) for 3 days; error bars represent variation among technical replicates; *n* = 3 independent experiments. **g** Representative graph of OCR of *Bap1*^*∆LysM*^ preOCs transduced with either vector or *HA-Pgc1β*. *Bap1*^*flox*^ serves as control. Error bar represents technical replicates; *n* = 2 independent experiments. **h** *Bap1*^*∆LysM*^ BMMs, transduced with *HA-Pgc1β* and exposed to M-CSF and RANKL (100 ng/mL) for 5 days, were stained for TRAP activity. The scale bar represents 500 µm; *n* = 2 independent experiments. Data represented as mean ± SEM; *P* values are shown by two-way ANOVA with Sidak’s multiple comparison testing (**a**–**d**, **f**–**h**) or two-sided student’s *t*-test (**e**). For **f**, *P* denotes *Bap*^*∆LysM*^ vector vs. *Bap*^*∆LysM*^ <*Bap1*^*wt*^. For **g,** *P* denotes *Bap*^*∆LysM*^ vs. *Bap*^*∆LysM*^ <*Pgc1β*.

ROS and mitochondrial metabolism have a bi-directional relationship, where ROS affects cellular metabolism and, reciprocally, mitochondrial respiration can impact ROS^19^; we thus investigated the effect of BAP1 on metabolism. As a first indicator of metabolic changes, we checked the mitochondrial copy number. Similar to ROS reduction, *mt-coII* is also significantly reduced in *Bap1*^***∆****LysM*^ preOCs (Fig. 6a). Additionally, expression of mitochondrial function genes and *Pgc1β*, which promotes mitochondrial biogenesis, is diminished in differentiated *Bap1*^***∆****LysM*^ preOCs relative to their floxed counterparts (Fig. 6b, c). To ascertain if cellular respiration is also disrupted in Bap1 deficient osteoclasts, we used Seahorse XF Analyzer to measure oxygen consumption rate (OCR) in osteoclast precursors. First, we titrated the concentration of the ATP synthase inhibitor, oligomycin (OA), and uncoupler, FCCP. Studies have indicated that accurate measurement of maximal respiratory capacity critically depends on the concentration of uncoupler, FCCP^20,21^. Moreover, excessive OA can also potentially cause energy failure to such an extent that it can impact subsequent respiration levels, Therefore, we assessed the optimal [FCCP] to [OA] that induces maximum respiration in osteoclasts. FCCP was sequentially added as indicated in Supplementary Fig. 6a. We conducted this experiment in the presence or absence of OA. We find 2 μM FCCP stimulates maximal respiration effectively without disrupting transport processes facilitated by Δψm (Supplementary Fig. 6a (black line). Importantly, when OCR was repeated in the presence of OA (blue line), no significant reduction in the respiratory capacity of osteoclast precursors was noted, suggesting that 1.5 μM OA dose does not negatively impact maximum respiratory capacity (Supplementary Fig. 6a, bottom panel). These inhibitor doses were used in subsequent OCR analyses of *Bap1*^*flox*^ and *Bap1*^*ΔLysM*^ osteoclast precursors. In line with our previous observations, *Bap1*^***∆****LysM*^ osteoclast precursors also exhibit decreased OCR (Fig. 6d). All parameters of OCR, namely, Basal respiration, ATP-coupled respiration and maximal respiration are impaired in osteoclasts lacking *Bap1*(Supplementary Fig. 6b). Extracellular acidification rate (ECAR) is reduced in the *Bap1*^*ΔLysM*^ preOCs (Supplementary Fig. 6c). Consistent with decreased cellular respiration, ATP production in the KO group is diminished (Fig. 6e). Importantly, *Bap1*^***∆****LysM*^ preOCs regain their respiratory capacity with reintroduction of *BAP1*^*WT*^ but not *BAP1*^*C91A*^ indicating mitochondrial respiration is regulated by BAP1 activity (Fig. 6f).

To determine whether the increasing mitochondrial number is sufficient to rescue *Bap1*^*ΔLysM*^ osteoclast respiration and function, we overexpressed *Pgc1β*, which promotes mitochondrial biogenesis, cytoskeletal organization, and bone degradative capcity^3^. *HA-Pgc1β* completely reestablishes mitochondrial biogenesis marker genes in *Bap1*^***∆****LysM*^ osteoclasts (Supplementary Fig. 6d, e) and normalizes respiratory capacity (Fig. 6g). Despite its mitochondrial normalizing capacity, *Pgc1β* reintroduction fails to rescue the cell’s crenated appearance (Fig. 6h). Thus, while *Bap1* deficiency suppresses osteoclast mitochondrial function, mitochondrial restoration alone, is insufficient to prevent the induced cytoskeletal defects.

### BAP1 regulates metabolic reprogramming in osteoclasts

**Fig. 7:**
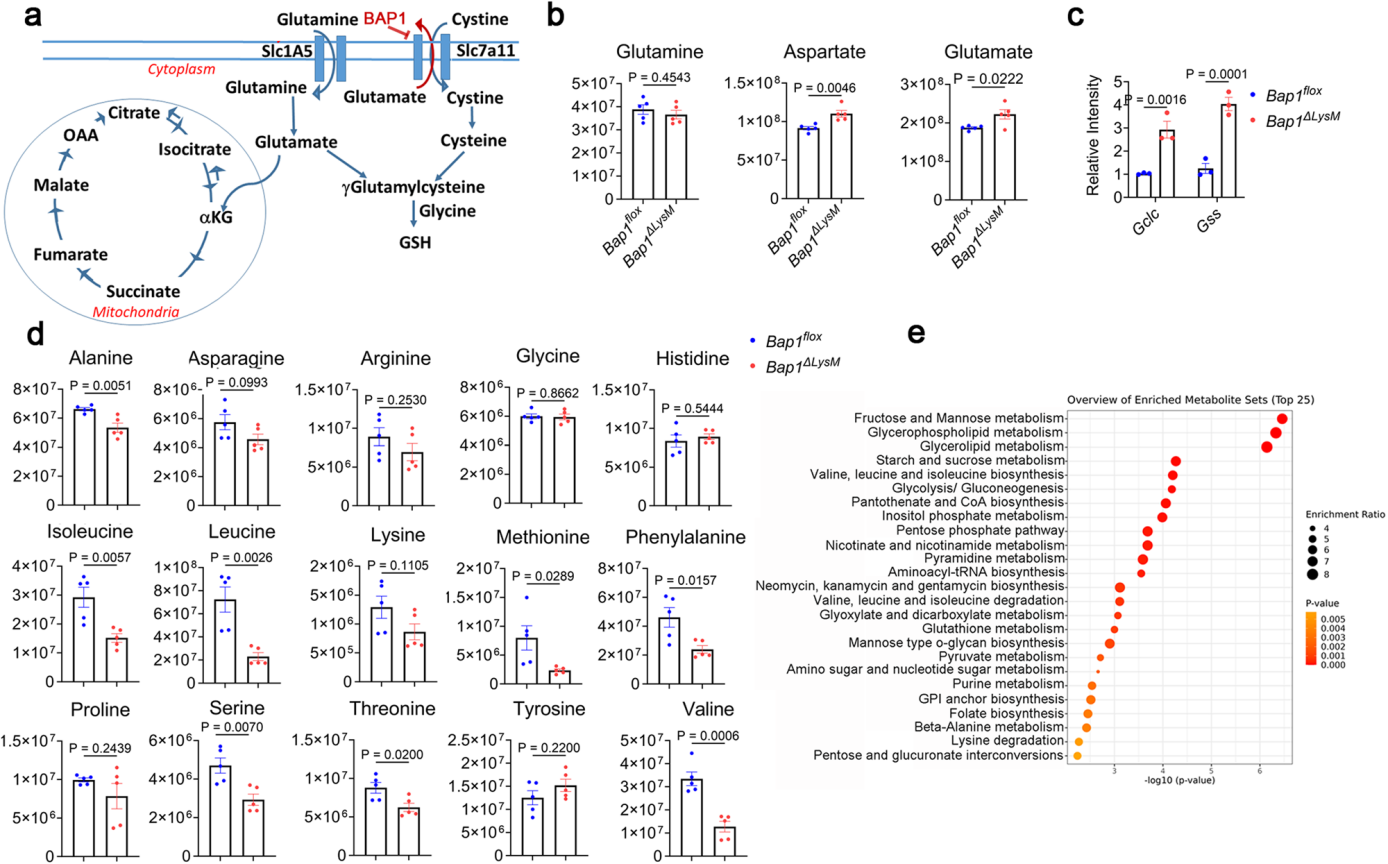
BAP1 regulates metabolic reprogramming in osteoclasts. **a** Schematic demonstrating the relationship between the TCA cycle, glutamine, and GSH synthesis. **b** Relative abundance of glutamine, aspartate, and glutamate in the cell determined by LC–MS analysis; *n* = 5 biologically independent samples from 20-week-old male mice. **c** Abundance of glutathione synthesis genes, *Gclc* and *Gss*, as determined by qPCR in preOCs; *n* = 3 biologically independent samples. **d** Relative abundance of amino acids in the cell by genotype; *n* = 5 biologically independent samples. **e** Pathway analysis using MetaboAnalyst for highly enriched metabolic pathways. Data represents mean ± SEM. *P* values are shown by two-way ANOVA with Sidak’s multiple comparison test (**c**) or two-sided student’s *t-*test (**b**, **d**). A global test was conducted for (**e**) to test the association between metabolites and outcome.

To gain insight into the relationship between decreased ROS, mitochondrial respiration, and osteoclast function, we performed LC/MS-based metabolomics. As shown in the schematic GSH biosynthesis is connected to the tricarboxylic acid (TCA) cycle via glutamate metabolism (Fig. 7a). Interestingly, glycolytic intermediates are elevated in *Bap1*^***∆****LysM*^ osteoclasts compared to controls (Supplementary Fig. 7a and Supplementary Data 1), while glycolysis genes are decreased in this group, suggesting glycolytic activity is downregulated (Supplementary Fig. 7b). Furthermore, while the TCA intermediate, aconitate accumulates in the cell, succinate and malate are decreased indicating inhibited respiration mirroring that of OCR (Supplementary Fig. 7a and Supplementary Data 1). Interestingly, aspartate and glutamate increased in *Bap1*^***∆****LysM*^ osteoclasts, while αKG did not change (Fig. 7b and Supplementary Data 1). It is likely that glutamate is utilized to generate GSH given the genes required to generate GSH namely glutamate cysteine ligase (*Gclc*) and glutathione synthetase (*Gss*) are upregulated in *Bap1*deficient osteoclasts (Fig. 7c). Moreover, glutathione S-transferase (GST) family genes that are necessary for conjugation of reduced glutathione (GSH) with various substrates are upregulated in these cells (Supplementary Fig. 7c). Thus, the data indicates that absence of BAP1 shifts glutamine toward glutathione synthesis and away from the TCA cycle.

Since TCA intermediates are also required for amino acid synthesis, we determined their relative abundance in the presence and absence of *Bap1*. Similar to RNAseq analysis, wherein amino acid metabolism is an enriched downregulated pathway, metabolomics reveals most amino acids, particularly BCAA, essential for osteoclast survival and function, are decreased (Fig. 7d) with a concomitant decline in protein synthesis (Supplementary Fig. 7d). In line with amino acid biosynthesis pathways, fructose and mannose metabolism pathways are significantly enriched (Fig. 7e). These data and RNAseq analysis suggest cellular metabolic reprogramming occurs with loss of *Bap1* which increases ROS and alters many aspects of cellular metabolism.

### Osteoclast function is restored by resupplementing TCA cycle metabolite

**Fig. 8:**
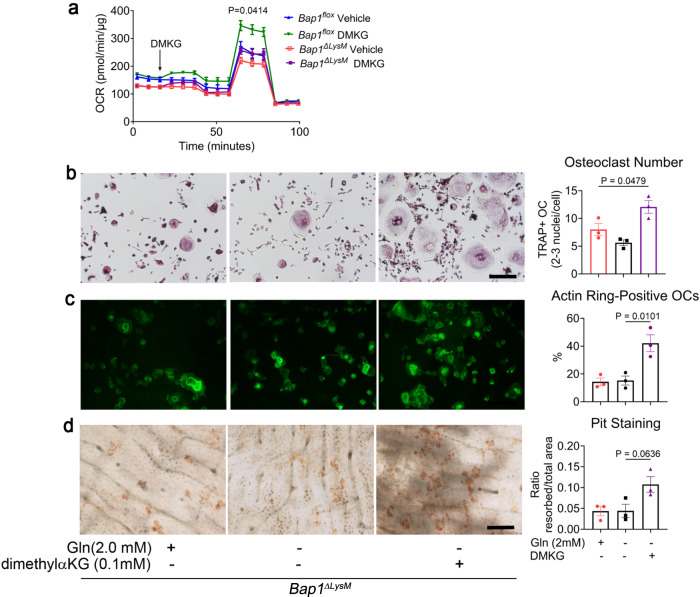
Osteoclast function is restored by resupplementing TCA cycle metabolite. **a** Representative OCR of *Bap1*^*flox*^ or *Bap1*^*∆LysM*^ preosteoclast treated acutely with vehicle or membrane-permeable α-KG analog (labeled as DMKG), followed by complex inhibitors. Error bars represent technical replicates; *n* = 2 biologically independent experiments from 16-week-old male mice. **b**–**d** *Bap1*^*∆LysM*^ osteoclast were cultured in either glutamine sufficient (Gln) or deficient (-Gln) αMEM medium in the presence of M-CSF RANKL (100 ng/ml) and DMKG; *n* = 3 independent experiments. **b** After 5 days, cells were stained for TRAP activity (scale bar represents 500 µm), **c** or cells were cultured on bone slices and stained with Alexa flour 488-Phalloidin to visualize and quantify actin rings (green color; scale bar represents 100 µm). **d** Following the removal of osteoclasts, resorption pits were visualized by wheat germ agglutinin-lectin and quantified (scale bar represents 100 µm). Data are represented as mean ± SEM; *P* values are shown by one-way ANOVA with Tukey’s multiple comparison testing (**a**–**d**). For Fig. **a**, *P* denotes the comparison between the *Bap1*^*∆LysM*^ vehicle vs. *Bap1*^*∆LysM*^ DMKG.

It is likely that the paucity of SLC7A11in WT osteoclasts maintain intracellular glutamate levels and promote its metabolism within the TCA cycle^22^. In contrast, the abundance of SLC7A11 in *Bap1*^***∆****LysM*^ osteoclasts either promotes glutamate utilization to generate GSH or is exported from the cell through the antiporter in exchange for cysteine^22^. Therefore, we asked if conversion of glutamate to α-ketoglutarate is sufficient to fuel the TCA cycle; and increase ROS, both important to maintain osteoclast function^23,24^. Supplementation with dimethyl α-ketoglutarate (dm-αKG), a cell-permeable form of α-ketoglutarate, increases mitochondrial respiration in *Bap1*^*flox*^ osteoclasts. Importantly, in *Bap1*^***∆****LysM*^ cells, dm-αKG normalizes the respiratory defect bringing OCR levels to those of vehicle-treated *Bap1*^*flox*^ cells (Fig. 8a). In addition, dm-αKG partially reverses *Bap1*^***∆****LysM*^ osteoclast’s crenated appearance, induces actin ring formation (Fig. 8b, c) and promotes resorption (Fig. 8d). Thus, our data describes a role for BAP1 in reprogramming metabolic events necessary for osteoclast function.

## Discussion

A range of essential molecules, from surface receptors such as RANK to co-stimulatory and transcription factors, are required for osteoclast function^10,25,26^. Many such transcription factors are controlled by epigenetic mechanisms such as histone modifications^1,27–29^. Although studies have associated epigenetic changes with osteoclast-specific transcription, their contributions to osteoclast function are incompletely understood. Here, we demonstrate that an epigenetic factor, BAP1, plays a crucial role in maintaining osteoclast function by reprogramming metabolic pathways. Its preferential effect on osteoclast activity over differentiation may be therapeutically relevant.

BAP1 is a component of a multiprotein complex that regulates cellular transcription by deubiquitinating histone, H2AK119ub1. While *Bap1* deletion in myeloid lineage cells does not change the expression of osteoclast differentiation markers, it alters the cell’s morphology producing a crenated appearance characteristic of cytoskeletal derangement^30^. Although *Bap1*^***∆****LysM*^ BMMs cultured on bone in the presence of RANKL and M-CSF become TRAP+, they do not form actin rings, establishing cytoskeletal disorganization. As a result of osteoclast dysfunction, mice bearing myeloid *Bap1* deletion develop high bone mass with reduced serum CTX. BAP1’s effect on osteoclast function is further confirmed by the fact that its elimination utilizing Cathepsin K cre, which despite targeting other tissues, is exclusively expressed in mature resorptive cells in the osteoclast lineage. Unexpectedly, we did not find differences in canonical signaling involving c-Src, Syk, and RAC that are required for the cytoskeletal organization even though the absence of any component of this pathway results in osteoclasts with contracted (“crenated”) appearance that fail to form actin rings or ruffled borders. Instead, our study revealed an epigenetic mechanism that links metabolic reprogramming to osteoclast function.

Bone resorption requires large ATP consumption that is supported by increased mitochondrial biogenesis^31^. However, the relationship between mitochondrial activity and osteoclast function is not linear. Nuclear-encoded protein NADH: ubiquinone oxidoreductase iron–sulfur protein 4 (NDUFS4) deficiency reduces bone resorption by suppressing mitochondrial complex I activity. Alternatively, disruption of the mitochondrial transcription factor A (*Tfam*) gene increases degradation^32,33^. Thus, the relationship between bone resorption and mitochondrial function is context-dependent, and the two processes may or may not be coupled despite pathway crossover.

In our study, a decline in mitochondrial function impedes resorption by *Bap1*-deficient osteoclasts. This phenomenon is reversed by restoring BAP1 deubiquitinase activity. Interestingly, restoration of mitochondrial function by overexpressing *Pgc1β* does not normalize cytoskeleton organization *Bap1* deficient cells. *Pgc1β*-induced oxidative phosphorylation also fails to restore the resorptive function of *Relb*^*−/−*^ or *Asxl2*^*−/−*^ osteoclasts, indicating essential contributions by other pathways, which likely include amino acid metabolism, glutaminolysis, fatty acid synthesis, and fatty acid oxidation^4,23,34^. Consistent with this hypothesis, RNAseq analysis of Bap1 deficient osteoclasts reveals the downregulation of genes involved in glucose and amino acid metabolism. Components of all these pathways, particularly lipids, such as phosphoinositides, regulate osteoclast morphology and act as second messengers and docking sites for multiple effectors^35–38^.

Our data also highlight a unique role for the amino acid transporter, SLC7A11, in regulating osteoclast function. We find that, unlike WT counterparts, *Bap1*^***∆****LysM*^ osteoclasts express abundant *Slc7a11*. Notably, ChIP qPCR data using BAP1 antibody indicates BAP1 binding to the *Slc7a11* promoter. Mutant constructs and H2AK119ub1 ChIP qPCRs also confirm epigenetic regulation of *Slc7a11* expression by BAP1. While individual ChIPs alone cannot preclude additional indirect mechanisms that may be involved in this gene regulation, our observation corroborates with other studies highlighting the complex role of BAP1 as an activator or repressor depending on the epigenetic landscape^12,39^. The paradoxical finding of how repressive H2AK119ub1 histone modification results in increased gene expression is intriguing and will require a detailed analysis of H2AK119ub1 in relation to other histone methylation marks.

Additionally, in order to fully interpret the effects of the *Bap1* complex on transcription in osteoclasts, it will be equally important to gain insight into how BAP1 interacts with other complex proteins such as ASXL1 and 2. While the unavailability of commercial antibodies against mouse ASXL1 or ASXL2 proteins limited our exploration in this regard, ChIPseq data from human cell lines shed light on their regulation of developmental and metabolic genes by these complex proteins^40–44^. Our data that BAP1 plays a role in regulating several metabolic pathways in osteoclasts also supports this concept.

Repression of *Slc7a11*, the cystine/glutamate xCT amino acid antiporter that antagonizes glutamate metabolism in osteoclasts, is one mechanism by which BAP1 regulates such pathways^12^. Since imported cystine is a necessary precursor of the antioxidant glutathione, an increase in the antiporter activity increases glutathione while decreasing intracellular ROS levels. This observation was consistent with a previous study by Hinoi et al. (2007), who also demonstrated the role of *Slc7a11* in regulating cellular ROS^45^. Exogenous^46,47^ or endogenous ROS^48,49^ stimulate the resorptive activity of osteoclasts. We find *Bap1* deletion-mediated decrease in osteoclast function reflects increased glutathione and, consequently, decreased ROS by SLC7A11. These data are consistent with previous reports wherein supplementation with *N*-acetylcysteine (NAC), a glutathione precursor, inhibits RANKL-induced osteoclastogenesis^16^. Importantly, inhibition of osteoclast function is prevented in *Bap1* deficient osteoclasts by knocking down *Slc7a11*. In addition to increasing cystine, SLC7A11 regulates the nutrient requirements of cultured cells by redirecting intracellular glutamate away from the TCA cycle^22^. Bypassing glutamate and supplementing osteoclasts with the glutamate metabolite α-ketoglutarate in *Bap1*^*∆LysM*^ osteoclasts partially normalizes their functional defects.

RNAseq and LC-MS highlight the enrichment of the BCAA pathway in *Bap1*^*ΔLysM*^ osteoclasts. Since previous studies have suggested a role of BCAA, particularly valine, in osteoclast maturation, other transcriptional and non-transcriptional Bap1 targets may also contribute to BAP1-mediated osteoclast function^13,50,51^. Stable isotope tracing studies may provide clarity on the role of metabolic reprogramming on osteoclast maturation and function.

Our data identify an epigenetic mechanism that links metabolic reprogramming to osteoclast function. This BAP1-initiated pathway includes regulation of mitochondrial metabolism and repressing *Slc7a11* expression. Because altered BAP1 and SLC7A11 each modulate osteoclast function, they present potential therapeutic targets for anti-resorptive drugs that do not diminish bone formation.

## Methods

### Reagents

Recombinant murine M-CSF was obtained from R&D Systems (Minneapolis, MN). Glutathione S-transferase (GST)-RANKL was expressed in our laboratory as described^52^. The source of reagents is as follows: Mito Stress Kit, 2-deoxyglucose, glucose, glutamine, and Seahorse assay medium was purchased from Agilent Technologies.

### Mice

All animals were housed in the animal care unit of Washington University School of Medicine, where they were maintained at 22 °C in a 12-h light–dark cycle according to guidelines of the Association for Assessment and Accreditation of Laboratory Animal Care. All mice used for experiments were healthy and had free access to water and food (Lab Diet, Rodent Diet 20 #5053). All mice were from in-house mating. Animal work was performed according to the policies of the Animal Studies Committee (ASC) at Washington University School of Medicine in St. Louis. Mice were analyzed under approved protocols (Protocol No. #23-0120) and were provided appropriate care while undergoing research which complies with the standards in the Guide for the Use and Care of Laboratory Animals and the Animal Welfare Act. For euthanasia, a CO_2_ gas chamber was used with the Smartbox setup with a flow meter calculated to deliver the appropriate flow rate (9 L/min). Both male and female mice were included in the study.

To generate *Bap1*^*∆LysM*^ or *Bap1*^∆CatK^ mice, *Bap1*^*flox/flox*^ mice (kindly provided by Prof. Anwesha Dey, Genentech Inc.) were mated with *LysM-Cre* or *CatK-Cre *mice to generate *Bap1*^*flox/wt*^*;LysM cre*^*+/*−^ or *Bap1*^*flox/wt*^;CatK^cre+/−^*mice respectively*. These mice were bred to obtain desired *Bap1*^*flox/flox*^ *LysMCre*^*+/−*^ (*Bap1*^*∆LysM*^) or *Bap1*^*flox/flox*^ *CatK*Cre^+/−^ (*Bap1*^*∆CatK*^) mice. *Bap1*^*fl/fl*^ littermate without Cre mice served as control.

### Macrophage isolation and osteoclast culture

All in vitro experiments were performed at least 3 times. Primary BMMs were prepared as described^52^ with slight modifications. Marrow was extracted from femora and tibiae of mice with α minimum essential medium (α-MEM) and cultured in α-MEM containing 10% inactivated fetal bovine serum, 100 IU/mL penicillin, and 100 μg/mL streptomycin (α-10 medium) with 1:10 of mMCSF producing cell line, CMG 14-12 condition media on petri-plastic dishes. Cells were incubated at 37 °C in 5% CO_2_ for 3 days and then washed with phosphate-buffered saline (PBS) and lifted with 1× trypsin/EDTA in PBS. A total of 1.2 × 10^4^ BMMs were cultured in 500 μL α-MEM containing 10% heat-inactivated fetal bovine serum with glutathione-S transferase–RANKL and 30 ng/mL of mouse recombinant macrophage colony-stimulating factor (M-CSF) in 48-well tissue culture plates, some containing sterile bovine bone slices. Cells were fixed and stained for tartrate-resistant acid phosphatase (TRAP) activity after 5 days in culture, using a commercial kit (Sigma 387-A; Sigma-Aldrich, St. Louis, MO). The images were captured using a Nikon Eclipse E400 (Melville, NY) upright microscope.

### Site-directed mutagenesis

Flag-HA-BAP1 was a gift from Wade Harper (Addgene plasmid # 22539; RRID:Addgene_22539)^53^. Mutant pDest-Flag-BAP1C91A constructs were generated by PCR mutagenesis using QuikChange II XL Site-Directed Mutagenesis Kit (Agilent) for amino acid substitutions according to the manufacturer’s instructions. All constructs were confirmed by DNA sequencing.

### Plasmids and retroviral transduction

Constructs expressing wild-type *Bap1(Bap1*^*WT*^*)* or mutant *Bap1(Bap1*^*C91A*^), *integrin β3* (generated in our laboratory^10^ *and HA-Pgc1β* (Kind gift from Deborah Veis, Washington University) in pMX retroviral vector were transfected into the Platinum E cells (PlatE) (Cell Biolabs, Cat No. RV101). The medium was changed on the next day, and the supernatant was then harvested, filtered, and infected the Day 2 BMMs in the presence of 1:10 CMG and 4 μg/ml polybrene (Sigma). Twenty-four hours later, the cells were selected with 1 μg/ml blastocidin for *integrin β3* and *Pgc1β* or 2 μg/ml puromycin for *Bap1*^*WT*^ or mutant *Bap1(Bap1*^*C91A*^*)* for at least 3 days before using as osteoclast precursors.

### Lentivirus infection

293-T cells (ATCC, Cat No. CRL3216) were transfected with either control or *crisprSlc7a11* (Millipore Sigma; MMPD0000046431) cloned in the vector (LV17) U6-gRNA:EF1a-Puro-2a-BFP together with a packaging plasmid (psPAX2 was a gift from Didier Trono (Addgene plasmid # 12260; RRID:Addgene_12260) and the envelope (pMD2.G was a gift from Didier Trono (Addgene plasmid # 12259; RRID:Addgene_12259) plasmid. After 48 h, a medium containing lentiviruses was collected and filtered. Macrophages obtained from 8- to 12-week-old male mice were infected with the virus for 24 h in the presence of 1:10 CMG and 10 µg/mL protamine (Sigma-Aldrich). Cells were selected in the presence of CMG and 1 μg/mL puromycin (Calbiochem) for 3 days before use as osteoclast precursors.

### TRAP staining assay

Cells were fixed and stained for TRAP activity after 5 days in culture, using a commercial kit (Sigma 387-A, St. Louis, MO).

### Actin ring formation assay

After 6 days of osteoclastic induction on bone slices, the osteoclasts were fixed and stained with Phalloidin (Alexa Fluor®488 Phalloidin, Cat No.8878, Thermo Scientific; dilution 1:60) at room temperature for 1 hour to visualize actin rings. ImageJ (https://imagej.nih.gov/ij/) was used to count the number of actin rings.

### Pit formation assay

After 6 days of culture, bone slices were incubated in 0.5 N NaOH for 30 s, and cells were scraped off using a cotton swab. Bone slices were then incubated with 20 μg/mL peroxidase-conjugated wheat germ agglutinin (Sigma) in PBS for 30 min, washed with PBS three times, and exposed to 3,3′-Diaminobenzidine tablets (Sigma; D4168) for 15 min before washing. ImageJ (https://imagej.nih.gov/ij/) was used to quantify the pit area.

### GTP-Rac1 detection assay

The GTP-Rac1 was detected using Active Rac1 Pull-Down and Detection Kit (Thermo Fisher Scientific, Cat No.16118). Briefly, day 3 pre-osteoclasts were starved for 3 h in α-MEM medium containing 2% FBS, lifted with 0.01% EDTA. Then the cells were seeded on the vitronectin-coated petri dish or left in 50 ml conical tubes in suspension at 37 °C for 30 min. Cells were lysed with Lysis/Binding/Wash Buffer. After quantification of the protein concentration, 500 μg cell lysates together with 20 μg GST-human Pak1-PBD were added to the pretreated glutathione resin for incubation at 4 °C for 1 h. The resin was washed 3 times, and protein was eluted using a 2× reducing sample buffer for western blotting.

### Histology and histomorphometry

The tibiae and femur of 20-week-old male mice were fixed with 10% neutral buffered formalin, followed by decalcification in 14% EDTA for 10 days, paraffin embedding, and TRAP staining. Images were collected using the NanoZoomer slide scanner Alafi Neuroimaging Laboratory. We analyzed one slide per mouse. To quantitate the TRAP-positive cells and determine how much of their surface area covered the bone (i.e., OcS/BS), we used the BioQuant Osteo2021 software (BioQuant Image Analysis Corporation, Nashville, TN). We carried out these measurements by defining a region of interest (ROI) for total tissue volume (TV; 1.5 mm below the growth plate and kept the same for all femurs analyzed) and bone volume (BV). We then chose a diagonal line type tool to define the bone surface (BS) and osteoclast surface (Oc.S). TRAP-positive osteoclasts were measured using the number tool on BioQuant Osteo2021 (BioQuant Image Analysis Corporation, Nashville, TN). Primary Indices were calculated from these: BV/TV, OcN/BS, OcS/BS. All measurements were done in a blinded fashion.

### Micro-computed tomography (μCT)

Micro-computed tomography (μCT) was done on the femurs of 20-week-old male and female mice or 1-year-old male mice. Bones were fixed in 10% neutral buffered formalin and then embedded in 2% agarose gel. The trabecular volume of the distal femoral metaphysis was measured using a Scanco μCT40 scanner (Scanco Medical AG, Bassersdorf, Switzerland), calibrated using a hydroxyapatite phantom. A threshold of 250 was used for evaluation for all male scans, and a threshold of 212 was used for evaluation for all female scans. Fifty slices were analyzed, starting with the first slice in which condyles and primary spongiosa were no longer visible. Trabecular bone was contoured to exclude the cortical bone, allowing cancellous bone volume/tissue volume (BV/TV) and BMD to be determined. vBMD was measured as mean values of everything within the volume of interest (mixture of bone and background). Since the scan was calibrated for bone, the mean value was represented in units of Hydroxyapatite density [mg HA/ccm].

### Serum CTX measurements

Serum CTX levels were measured by ELISA (Biomedical Technologies Inc.) according to the manufacturer’s protocol.

### Calcein double labeling

Two-month-old *Bap1*^*flox*^ or *Bap1*^*ΔLysM*^ male littermates were injected intraperitoneally with calcein (Sigma) (7.5 mg/kg of body weight) on days 6 and 2 before sacrifice. Non-decalcified histological sections of the femur were analyzed using BioQuant Osteo2021 (BioQuant Image Analysis Corporation, Nashville, TN).

### Oxygen consumption and ECAR analysis

OCR and ECAR were measured using a Seahorse XFe96 Extracellular Flux Analyzer. Agilent Seahorse XF Cell Mito Stress Test Kit (Cat No. 103015-100) was used for the assays. Briefly, BMMs were seeded in a Seahorse 96-well cell culture microplate at a density of 6 × 10^4^ cells/well. The cells were osteoclast-induced with 100 ng/ml GST-RANKL and 30 ng/ml M-CSF for 3 days. On the day of the assay, culture media was changed to unbuffered XF DMEM (pH7.4) (Agilent Technologies, Cat No 103575-100) supplemented with 10 mM glucose, 200 ng/ml GST-RANKL and 100 ng/ml M-CSF, 1 mM pyruvate and 2 mM glutamine. The cells were equilibrated for 1 h at 37 °C in a CO_2_-free incubator. The OCR measurement cycle consisted of 1.5-min mixing and a 5-min measurement of the oxygen level. Testing of mitochondrial function was initiated by three baseline OCR measurement cycles. These were followed by the sequential injection of oligomycin (1 μM final concentration), Trifluoromethoxy carbonyl cyanide phenylhydrazone (FCCP) (2 μM), and a mixture of rotenone (1 μM) and antimycin A (1 μM) with one OCR measurement cycle in between each injection and two final measurement cycles. For the FCCP dose titration, a mito stress test was conducted as described above, but with the sequential addition of increasing FCCP dose (0.5 μM followed by 1.5 μM) in the presence or absence of OA injection. OCR was normalized to μg protein and calculated by the Seahorse XFe96 software, Wave 2.6.1. Based on the OCR measurements, mitochondrial respiration parameters were calculated as follows: basal respiration, non-mitochondrial respiration (minimum measurement after rotenone/antimycin injection) was subtracted from the last measurement obtained before oligomycin injection; maximal respiration, measurement obtained after FCCP injection; oxygen consumption associated with ATP-coupled respiration, measurement after oligomycin injection was subtracted from the last measurement before oligomycin injection; spare respiratory capacity, basal respiration subtracted from maximal respiration. Measurement of ECAR was also calculated during the Mitostress test by the Seahorse XFe96 software, Wave.

### RNA extraction and quantitative qPCR

RNA from cultured cells was isolated and purified using the RNeasy RNA purification kit (Qiagen); RLT lysis buffer was supplemented with β-mercaptoethanol (1%). Purified RNA was treated with DNase I (Invitrogen) before reverse transcription. cDNA was synthesized from RNA (1 μg) using iScriptTM cDNA Reverse Transcription kit (Bio-Rad) per manufacturer’s instructions. Real-time PCR was performed using the SYBR Green Master Mix kit and gene-specific primers. Quantitative PCR was performed on the ABI PRISM 7500 sequence detection system (Applied Biosystems, Foster City, CA). All reactions were performed in triplicates, and relative mRNA levels were calculated by the comparative threshold cycle method using GAPDH as an internal control. Oligonucleotide sequences are provided in Supplementary Table 1.

### Immunoblotting

Cultured cells were washed twice with ice-cold PBS and lysed in radioimmune precipitation assay (RIPA) buffer containing 20 mm Tris-HCl, pH 7.5, 150 mm NaCl, 1 mm EDTA, 1 mm EGTA, 1% Triton X-100, 2.5 mm sodium pyrophosphate, 1 mm β-glycerophosphate, 1 mm Na3VO4, 1 mm NaF, and 1× protease inhibitor mixture (Roche Applied Science). After incubation on ice for 10 min, cell lysates were clarified by centrifugation at 15,000 rpm for 10 min. For suspension adhesion assay, day 3 pre-osteoclasts were starved for 3 h in α-MEM medium containing 2% FBS, followed by lifting cells with 0.01% EDTA. Cells were seeded on the vitronectin-coated petri dish or left in 50 ml conical tubes in suspension at 37 °C for 30 min.

Forty micrograms of total lysates were subjected to 8–12% sodium dodecyl sulfate-polyacrylamide gel electrophoresis and transferred onto PVDF membranes. Filters were blocked in 0.1% casein in PBS for 1 h and incubated with primary antibodies at 4 °C overnight, followed by probing with fluorescence-labeled secondary antibodies (Jackson ImmunoResearch Laboratories). Proteins were detected with the Odyssey Infrared Imaging System (LI-COR Biosciences). Antibodies used in these assays are provided in Supplementary Table 2.

### RNA sequencing and analysis

Total RNA integrity was determined using an Agilent Bioanalyzer. Library preparation was performed with 5–10 µg of total RNA with a Bioanalyzer RIN score greater than 8.0. Ribosomal RNA was removed by poly-A selection using Oligo-dT beads (mRNA Direct kit, Life Technologies). mRNA was then fragmented in reverse transcriptase buffer and heated to 94° for 8 min. mRNA was reverse transcribed to yield cDNA using SuperScript III RT enzyme (Life Technologies, per manufacturer’s instructions) and random hexamers. A second strand reaction was performed to yield ds-cDNA. cDNA was blunt-ended, had an A base added to the 3′ ends, and then had Illumina sequencing adapters ligated to the ends. Ligated fragments were then amplified for 12–15 cycles using primers incorporating unique dual index tags. Fragments were sequenced on an Illumina NovaSeq-6000 using paired-end reads extending 150 bases.

Basecalls and demultiplexing were performed with Illumina’s bcl2fastq software and a custom Python demultiplexing program with a maximum of one mismatch in the indexing read. RNA-seq reads were then aligned to the Ensembl release 76 Mus musculus primary assembly with STAR version 2.5.1a^54^. Gene counts were derived from the number of uniquely aligned unambiguous reads by Subread:featureCount version 1.4.6-p5^55^. Isoform expression of known Ensembl transcripts was estimated with Salmon version 0.8.2^56^. Sequencing performance was assessed for the total number of aligned reads, total number of uniquely aligned reads, and features detected. The ribosomal fraction, known junction saturation, and read distribution over known gene models were quantified with RSeQC version 2.6.2^57^.

All gene counts were then imported into the R/Bioconductor package EdgeR^58^, and TMM normalization size factors were calculated to adjust for samples for differences in library size. Ribosomal genes and genes not expressed in the smallest group size minus one sample greater than one count per million were excluded from further analysis. The TMM size factors and the matrix of counts were then imported into the R/Bioconductor package Limma^59^. Weighted likelihoods based on the observed mean-variance relationship of every gene and sample were then calculated for all samples, and the count matrix was transformed to moderated log 2 counts-per-million with Limma’s voomWithQualityWeights^60^. The performance of all genes was assessed with plots of the residual standard deviation of every gene to their average log count with a robustly fitted trend line of the residuals. Differential expression analysis was then performed to analyze for differences between conditions, and the results were filtered for only those genes with Benjamini-Hochberg false-discovery rate adjusted *p*-values less than or equal to 0.05.

For each contrast extracted with Limma, global perturbations in known Gene Ontology (GO) terms, MSigDb, and KEGG pathways were detected using the R/Bioconductor package GAGE^61^ to test for changes in expression of the reported log 2 fold-changes reported by Limma in each term versus the background log 2 fold-changes of all genes found outside the respective term. The R/Bioconductor package heatmap3^62^ was used to display heatmaps across groups of samples for each GO or MSigDb term with a Benjamini-Hochberg false-discovery rate adjusted *p*-value less than or equal to 0.05. Perturbed KEGG pathways where the observed log 2 fold-changes of genes within the term were significantly perturbed in a single-direction versus background or in any direction compared to other genes within a given term with p-values less than or equal to 0.05 were rendered as annotated KEGG graphs with the R/Bioconductor package Pathview^63^.

To find the most critical genes, the Limma voomWithQualityWeights transformed log 2 counts-per-million expression data was then analyzed via weighted gene correlation network analysis with the R/Bioconductor package WGCNA^64^. Briefly, all genes were correlated across each other by Pearson correlations and clustered by expression similarity into unsigned modules using a power threshold empirically determined from the data. An eigengene was then created for each de novo cluster, and its expression profile was then correlated across all coefficients of the model matrix. Because these clusters of genes were created by expression profile rather than known functional similarity, the clustered modules were given the names of random colors, where gray is the only module that has any pre-existing definition of containing genes that do not cluster well with others. These de-novo clustered genes were then tested for functional enrichment of known GO terms with hypergeometric tests available in the R/Bioconductor package clusterProfiler^65^. Significant terms with Benjamini-Hochberg adjusted p-values less than 0.05 were then collapsed by similarity into clusterProfiler category network plots to display the most significant terms for each module of hub genes in order to interpolate the function of each significant module. The information for all clustered genes for each module was then combined with their respective statistical significance results from Limma to determine whether or not those features were also found to be significantly differentially expressed. The RNAseq data generated in this study have been deposited in the Gene Expression Omnibus database under accession code 231819.

### Chromatin immunoprecipitation (ChIP) assay

For chromatin fixation, 6 × 10^6^ million cells were plated in 150 mm tissue culture plates (MCSF + 100 ng/ml RANKL) for 3 days. Formaldehyde was added directly to cell culture media for 10 minutes at room temperature such that the final concentration was 1%. Cross-linking was quenched by the addition of glycine to a final concentration of 0.125 M. Cells were washed 3 times with PBS and scraped off the plates in 1 ml of PBS containing protease inhibitors (Thermo Scientific, Cat No. 78443). Cells were centrifuged at 1000 × *g* for 5 min at 4 °C. The pellet was resuspended and lysed in 300 ul of SDS Lysis Buffer (50 mM [tris(hydroxymethyl)aminomethane [Tris]–HCl pH 8], 10 mM [EDTA], 1.0% [sodium dodecyl sulfate [SDS]], and protease inhibitors) for 20 minutes on ice. Cells were chromatin sheared using Bioruptor (6 cycles, 10 minutes at high speed per cycle). The samples were centrifuged to pellet the cellular debris at 15,000 × *g*. Ten percent of the cells were collected in a separate tube to be used as input control. The supernatant was divided equally between immunoprecipitation samples which also included isotype control.

For immunoprecipitation, 60 μL of magnetic protein A/G beads were used. The beads were washed thrice with PBS containing 0.02% tween 20. After the final wash, beads were resuspended with the antibody overnight at 4 °C (antibody details used for ChIP assays are provided in supplemental table 2). Sheared DNA was incubated with the bead-antibody slurry. The next day, DNA-protein complexes were washed in low salt buffer (0.1% [SDS], 1%[Triton X100], 2 mM [EDTA], 20 mM [Tris-HCl pH 8.0], 150 mM [NaCl]) followed by high salt buffer (0.1% [SDS], 1% [Triton X100], 2 mM [EDTA], 20 mM [Tris-HCl pH 8.0], 500 mM [NaCl]), LiCl buffer (0.25 M [LiCl], 1% [nonidet P-40], 1% [deoxycholate], 1 mM [EDTA], 10 mM [Tris-HCl pH 8.0]), and Tris-EDTA buffer (Sigma Aldrich, Cat No. 574793). DNA was eluted by adding 250 μL of elution buffer (1% [SDS], 0.1 M [NaHCO3]). Samples and inputs were de-cross-linked and cleaned for quantitative polymerase chain reaction (qPCR) analysis.

### ATP quantification assay

ATP was quantified using an ATP Determination Kit (Invitrogen, A22066). Briefly, the reaction mixture was added to the 96-well cell culture plate (100 μl/well), which contains the cultured cells or standard solution. After 30 min of incubation in the dark, the plate was read using a luminescence detector. ATP concentrations were calculated using the standard curve.

### GSH assay

GSH was calculated using a GSH-Glo assay kit (Promega, V6911). Briefly, cells were plated in 96-well opaque plates at a density of 5000 cells/well in the presence of 1:10 CMG and 100 ng/ml of RANKL for 3 days. On the day of the assay, media was removed from the wells. In total, 100 μl of prepared 1× GSH-Glo™ Reagent was added to each well after briefly mixing; the plate was incubated at room temperature for 30 min. In total, 100 μl of reconstituted Luciferin Detection Reagent was added to each well of a 96-well plate. Luminescence was measured using a luminometer. GSH concentrations were calculated using the standard curve.

### ROS assay

Cells were plated in 96-well opaque plates at a density of 5000 cells/well in the presence of 1:10 CMG and 100 ng/ml of RANKL for 3 days. On the day of the assay, media was removed from the wells. cells were washed and incubated in the dark for 15 min with α-MEM lacking phenol red in the presence or absence of H_2_O_2_ (50 μM). In total, 5 μM DCFH-DA (Sigma Aldrich, Cat No. 35845) was added in the last 10 min of incubation. DCF (2′,7′-dichlorofluorescein) fluorescence was measured with an excitation wavelength of 488 nm and emission at 515–540 nm.

### Mitochondrial membrane potential

Cells were plated in 384-well opaque plates at a density of 1000 cells/well in the presence of 1:10 CMG and 100 ng/ml of RANKL for 5 days. For mitochondrial staining, cells were incubated with 100 μM TMRM dye (Thermos Scientific, Cat No. T668) diluted in DMEM assay media (Agilent Technologies, Cat No 103575-100) in the presence of 50 μM verapamil. TMRM was incubated for 120 min at 37 °C without washing and then challenged with 2 µM FCCP (Sigma Aldrich, Cat No. C2920). Live cell images were acquired, and absorbance was quantified using Agilent Biotek Cytation.

### LC–MS analysis

Cells were quenched with cold LC/MS-grade methanol, then scraped and transferred to Eppendorf tubes. Samples were dried in a SpeedVac. The samples were then reconstituted in 1 mL of cold methanol:acetonitrile:water (2:2:1) and subjected to three cycles of vortexing, freezing in liquid nitrogen, and 10 min of sonication at 25 °C. Samples were stored at −20 °C overnight and then centrifuged for 10 min at 14,000 × *g*, and 4 °C. Supernatants were transferred to new tubes and dried by a SpeedVac. The protein abundance of each sample was determined by using BCA. A quantity of 1 μl of acetonitrile:water (2:1) per each 2.5 μg of protein was used. Samples were subjected to two cycles of vortexing and 10 min of sonication at 25 °C. Next, samples were centrifuged for 10 min at 14,000 × *g* and 4 °C, transferred supernatant to LC vials, and stored at −80 °C until MS analysis.

Ultra-high-performance LC (UHPLC)/MS was performed with a Thermo Scientific Vanquish Flex UHPLC system interfaced with a Thermo Scientific Orbitrap ID-X Tribrid Mass Spectrometer (Waltham, MA). Hydrophilic interaction liquid chromatography (HILIC) separation was accomplished by using a HILICON iHILIC-(P) Classic column (Tvistevagen, Umea, Sweden) with the following specifications: 100 × 2.1 mm, 5 μm. Mobile-phase solvents were composed of A = 20 mM ammonium bicarbonate, 0.1% ammonium hydroxide, and 2.5 μM medronic acid in water:acetonitrile (95:5) and B = acetonitrile:water (95:5). The column compartment was maintained at 45 °C for all experiments. The following linear gradient was applied at a flow rate of 250 μL min^−1^: 0–1 min: 90% B, 1–12 min: 90-35% B, 12–12.5 min: 35-25% B, 12.5–14.5 min: 25% B. The column was re-equilibrated with 20 column volumes of 90% B. The injection volume was 2 μL for all experiments. LC/MS data were processed and analyzed with the open-source Skyline software^66^, decoID^67^, and publicly available MetaboAnalyst^68^. All the metabolomic data has been included in Supplementary Data 1.

### Statistics

Statistical significance was determined using multiple comparisons in a one-way or two-way analysis of variance (ANOVA) test or with unpaired nonparametric Student *t*-test when only two groups were present, using GraphPad Prism v10 built-in statistical analysis (GraphPad Software Inc., La Jolla, CA). *P* < 0.05 was considered significant. All quantitative reverse transcription-PCR and qPCR data were expressed as mean from at least three independent biological experiments with at least three technical replicates.

### Reporting summary

Further information on research design is available in the Nature Portfolio Reporting Summary linked to this article.

### Supplementary information

Supplementary Information

Description of Additional Supplementary Files Document

Supplementary Data 1

Reporting Summary

### Source data

Source Data

## Data Availability

The RNA-seq data generated in this study have been deposited in the Gene Expression Omnibus database under the accession code GSE231819. The metabolomic data in this study is provided as Supplementary Data 1 and accessible on the Metabolomics Workbench (https://www.metabolomicsworkbench.org/) using study ID ST002850. Source data are provided as a Source Data file. Source data are provided in this paper.
