# Supplementary material for: BAP1 promotes osteoclast function by metabolic reprogramming
Source: Nat Commun. 2023 Sep 22;14:5923. doi: 10.1038/s41467-023-41629-4 (PMC10516877; doi:10.1038/s41467-023-41629-4)

SUPPLEMENTARY INFORMATION

BAP1 promotes osteoclast function by metabolic reprogramming.

Nidhi Rohatgi, Wei Zou, Yongjia Li, Kevin Cho, Patrick L. Collins, Eric Tycksen,
Gaurav Pandey, Carl J. DeSelm, Gary J. Patti, Anwesha Dey and Steven L. Teitelbaum

Supplementary Figures

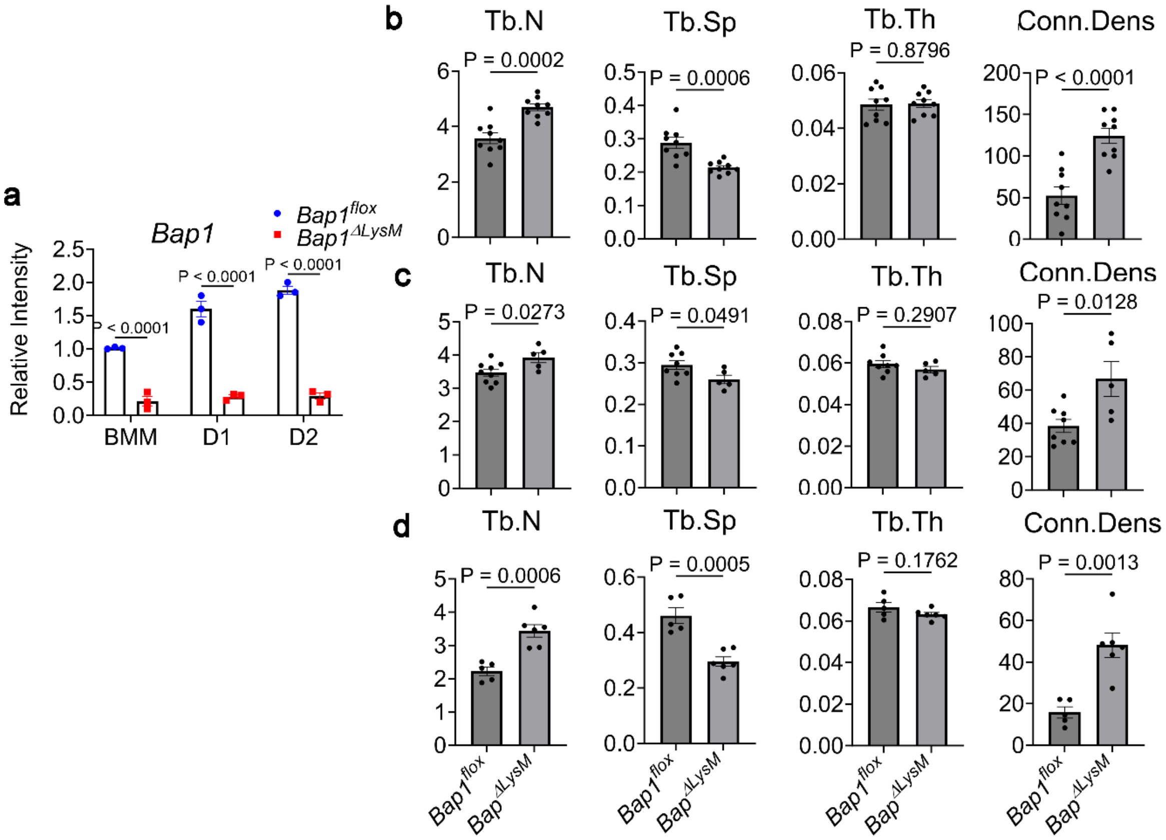

Supplementary Fig. 1: *Bap1$^{\Delta LysM}$* Mice Are Osteopetrotic. a) *Bap1^{flox}* and *Bap1$^{\Delta LysM}$* BMMs derived from 8-week old mice were exposed to MCSF $\pm$ RANKL (100 ng/ml) for indicated time. *Bap1* mRNA expression was determined by qPCR analysis. Day 2 untreated BMMs serve as control. **b-d** μCT analysis of femurs of (**b**) 20 week old males (n=9 biological samples); (**c**) 20 week old females (*Bap1^{flox}* n=6 and *Bap1$^{\Delta LysM}$* n=5) (**d**)1 year old male mice (*Bap1^{flox}* n = 5 and *Bap1$^{\Delta LysM}$* n=6 mice). Conn-Dens, connectivity density, normed by TV; TbN, trabecular number; TbSp, trabecular separation; TbTh, trabecular thickness. Data represent mean $\pm$ SEM. *P* values shown are determined by two-way ANOVA with Sidak multiple comparison testing (**a**) or two-sided Student’s *t*-test (**b-d**). Source data are provided as Source Data file.

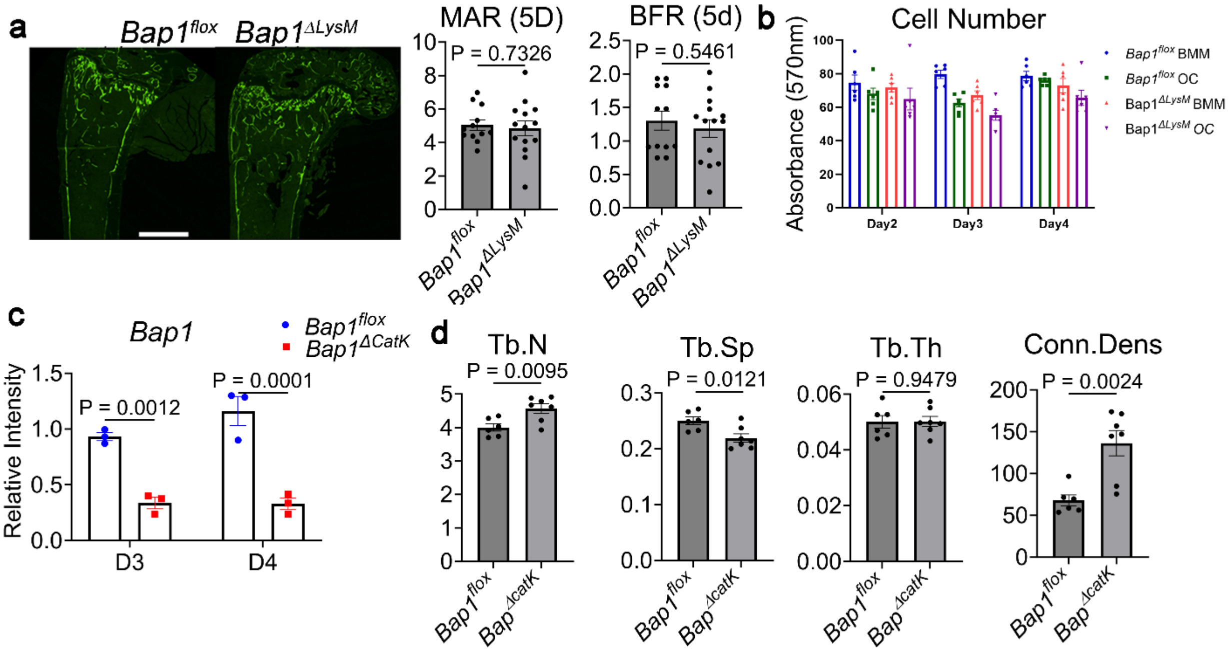

Supplementary Fig. 2: Bap1 Regulates Osteoclast Function. a Double labelling in 8 week old *Bap1*^{flox} and *Bap1*$^{\Delta LysM}$ male mice (*Bap1*^{flox} n=12 and *Bap1*$^{\Delta LysM}$ n=14 biologically independent samples).Scale Bar represents 1mm. **b** *Bap1*^{flox} and *Bap1*$^{\Delta LysM}$ BMMs cultured with M-CSF and RANKL (100 ng/mL) for days as indicated. Alamar Blue assay was done to measure cell numbers (n=5-6 biologically independent samples). **c** Gene expression analysis of *Bap1* deletion in 20-week old *Bap1*$^{\Delta catK}$ mice (n=3 independent experiments). **d** µCT analysis of femurs of 20-week old *Bap1*^{flox} and *Bap1*$^{\Delta catK}$ males (*Bap1*^{flox} n = 6 and *Bap1*$^{\Delta LysM}$ n=7 biologically independent samples). Data represents mean $\pm$ SEM. *P* values shown are determined by two-sided Student's *t* test (**a, d)** or two-way ANOVA with Sidak's multiple comparison testing **(b, c)**. Source data are provided as a Source Data file.

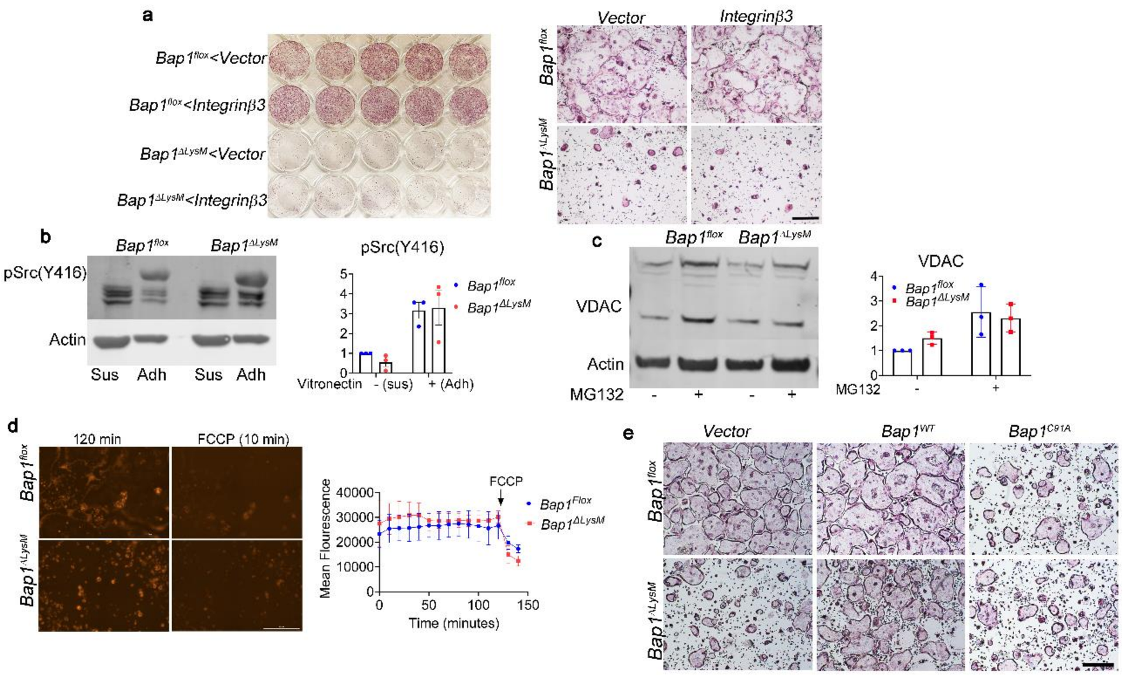

Supplementary Fig. 3: Bap1 epigenetic activity modulates osteoclast function. a Representative image of whole plate (left) and TRAP stained microscopic image (right) of *Bap1*^{flox} and *Bap1*$^{\Delta LysM}$ osteoclast derived from 8-12 week old male mice transduced with *integrin β3* or *vector* (n=2 independent experiments). Scale bar represents 500µm. **b** Day 3 *Bap1*^{flox} and *Bap1*$^{\Delta LysM}$ preOCs derived from 8-12 week old male mice were cultured for 3h in serum-free medium. The cells were plated on vitronectin-coated petri-dishes or maintained in suspension for 30 min. Total cell lysate was collected and pSrc(Y416) and Actin were determined by immunoblot; n=3 independent experiments. **c** *Bap1*^{flox} and *Bap1*$^{\Delta LysM}$ preOCs were cultured for 30 min with proteasome inhibitor, 5 µM MG132 or DMSO as control. Cell lysates were immunoprecipitated (IP) with ubiquitin antibody and analyzed by Western blotting (WB) with VDAC antibody; n=3 independent experiments. **d** Mitochondrial membrane potential (MMP) was measured in live cells for 2h followed by FCCP treatment. Representative image of TMRM staining in osteoclast (left) scale bar represents 200µm; quantification of MMP over 2h

(right) (n=2). **e** Representative image of $Bap1^{flox}$ and $Bap1^{\Delta LysM}$ osteoclast transduced with *vector*, $Bap1^{WT}$ or $Bap1^{C91A}$ constructs after which cells were stained for TRAP activity (n=3 independent experiments). Scale bar represents 500μm. Data represents mean $\pm$ SEM. Statistics conducted by two-way ANOVA with Sidak's multiple comparison testing (**c,d,e**). No significance was noted in comparison to their respective controls. Source data are provided as Source Data file.

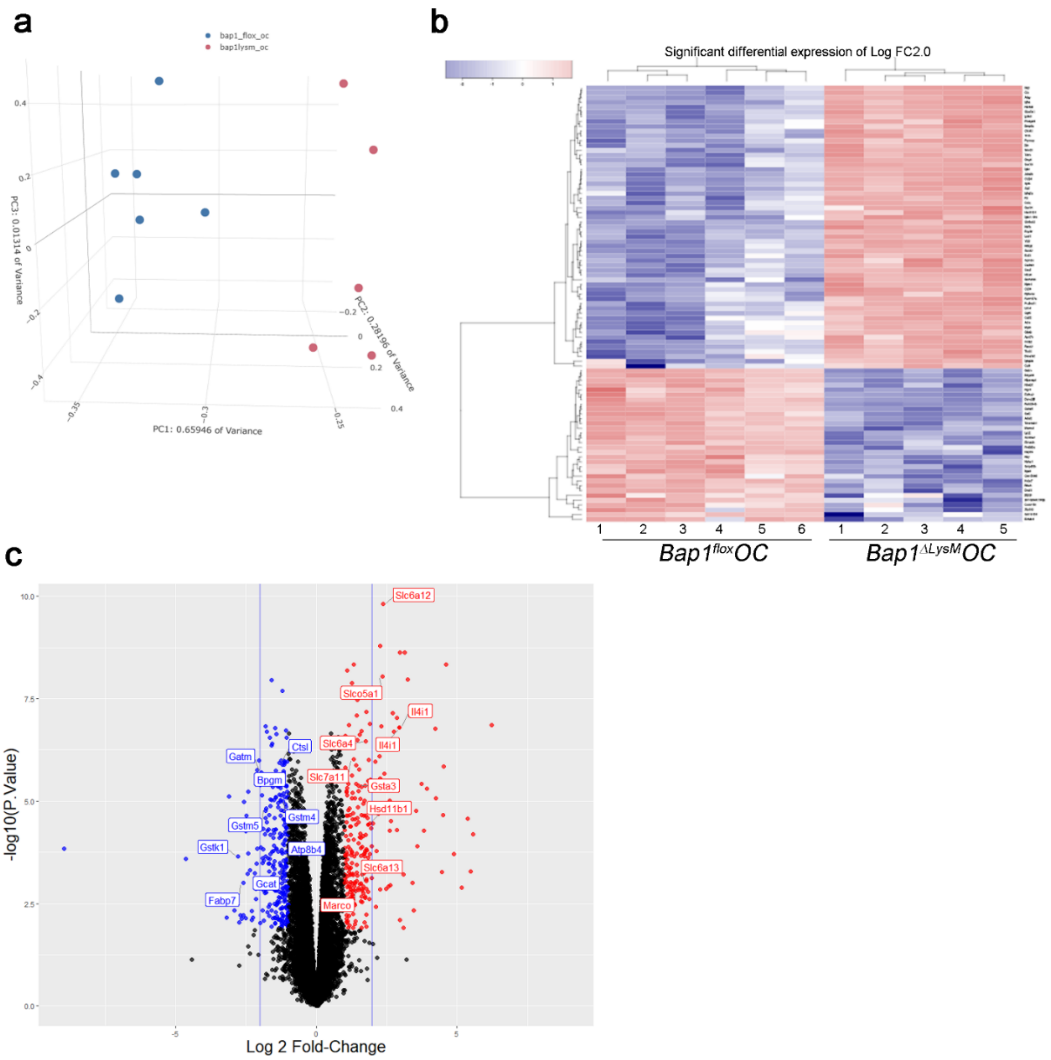

Supplementary Fig. 4: Bap1 regulates osteoclast transcriptional program. a PCA plot demonstrating separation by genotype. **b** Heatmap of 50 most upregulated (red) or downregulated (blue) genes during RNA-seq of mRNAs of *Bap1*^{flox} and *Bap1*$^{\Delta LysM}$ preOCs based on the Log FC value. **c** Volcano plots of RNAseq data to visualize genes (data points) that are being identified as differentially expressed in *Bap1*$^{\Delta LysM}$ osteoclast compared with *Bap1*^{flox}. The red and blue dots represent genes with at least 1.5-fold increase or decrease in *Bap1*$^{\Delta LysM}$ respectively. Candidate genes involved in metabolism are identified in blue and solute carriers identified in red.

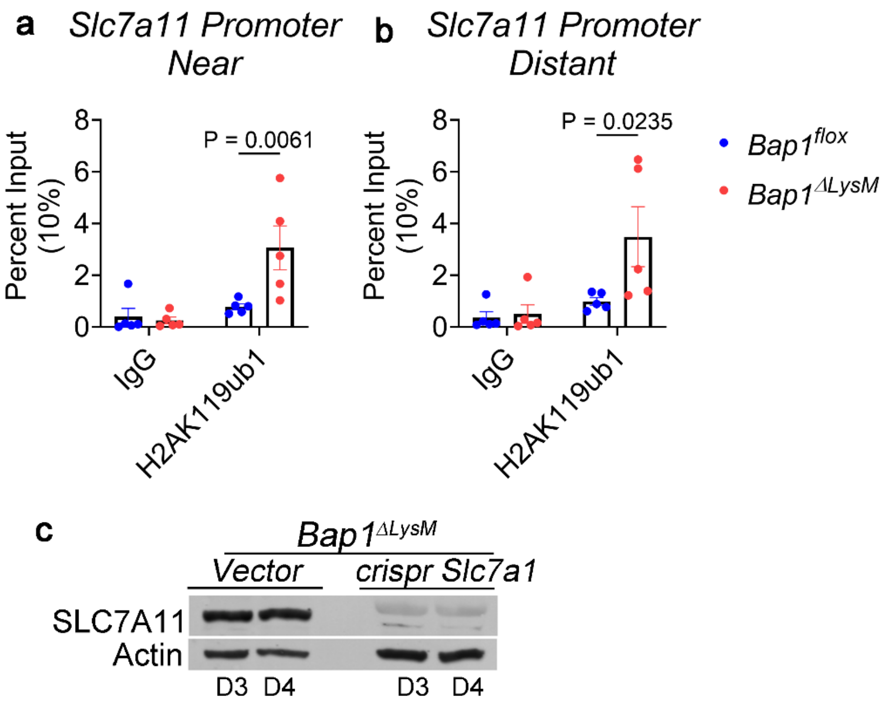

Supplementary Fig. 5: Bap1 regulates *Slc7a11* expression. a-b ChIP-qPCR showing H2Aub binding on different *Slc7a11* promoter regions (near and distant) in 16-20 week old *Bap1*$^{\Delta LysM}$ and *Bap1*^{flox} preOCs (n = 3 biologically independent samples). **c** Representative blot for SLC7A11 protein expression in *Bap1*$^{\Delta LysM}$ BMMs knockdown with *Slc7a11 and* exposed to M-CSF and RANKL (100 ng/ml) for 3 and 4 days (n=2 biologically independent samples). Data represents mean $\pm$ SEM. *P* values shown are determined by two-way ANOVA with Sidak's multiple comparison test used for statistical analysis (**a,b**). Source data are provided as a Source Data file.

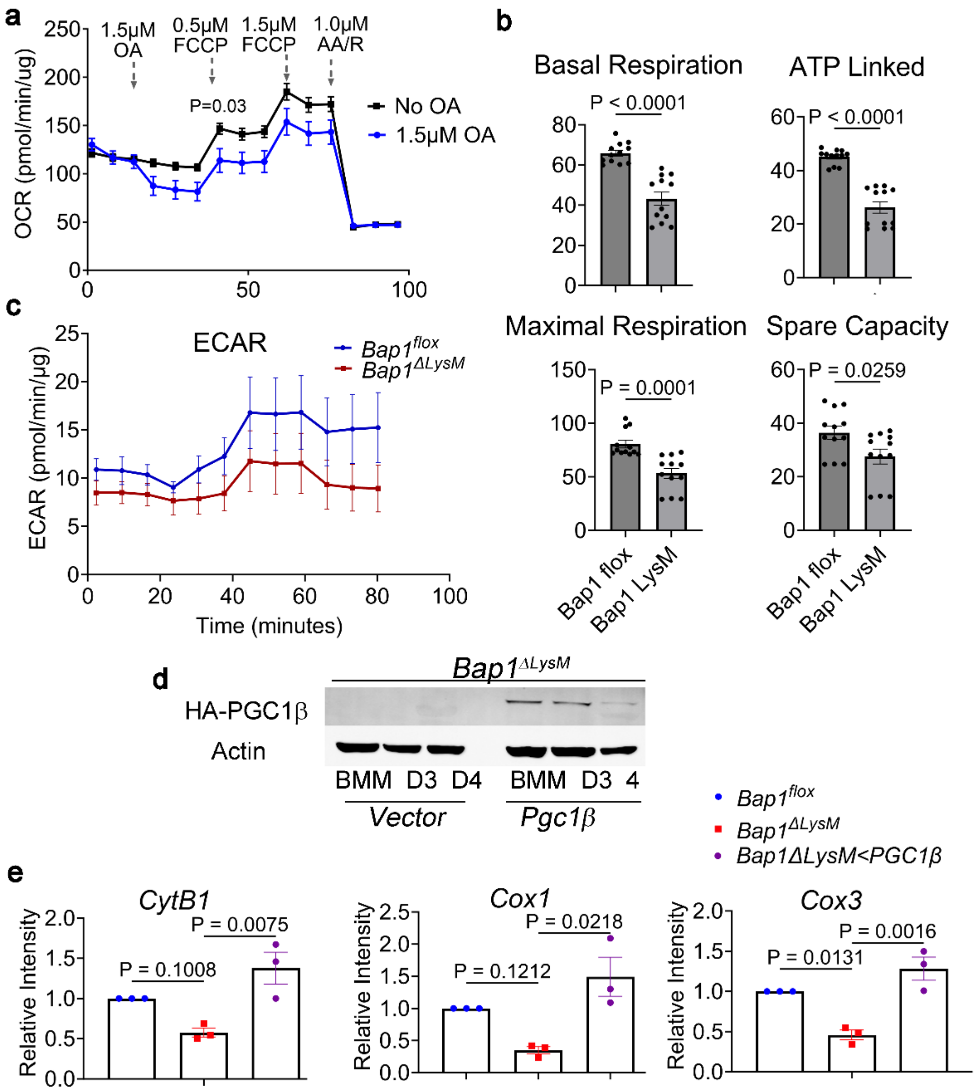

Supplementary Fig. 6: Bap1 regulates osteoclast mitochondrial respiration. a Oxygen consumption profiles for WT osteoclast precursors. 6.0×10^4 cells/well were plated in XF96 plates and grown for 3 days in presence of 100ng/ml RANKL. On the day of the assay, culture media was changed to unbuffered DMEM (pH7.4) supplemented with 10 mM glucose, 200 ng/ml GST-RANKL and 100 ng/ml M-CSF, 1mM pyruvate and 2 mM glutamine. Arrows indicate sequential additions of oligomycin (1.5µM, except for black line group), two sequential pulses of FCCP [FCCP]= 0.5µM followed by 1.5 µM (2.0µM final), and rotenone with antimycin A (1.0 µM) (n=2 independent experiments). **b** Quantification of OCR parameters of

$Bap1^{flox}$ and $Bap1^{\Delta LysM}$ preOCs. **c** Extracellular acidification rate (ECAR) measured during mito stress test assay (n=4 independent experiments). **d** and **e** *$Bap1^{\Delta LysM}$* BMMs transduced with *Pgc1β* or *vector*, were exposed to M-CSF and RANKL. **d** Abundance of HA-PGC1β determined by immunoblot and (**e)** qPCR determined mitochondrial biogenesis markers mRNA (n=2 independent experiments). Data represents mean ± SEM. *P* values shown are determined by two-way ANOVA with Sidak's multiple comparison test (**a, c, e)** or two sided Student *t* test (**b**). Source data are provided as Source Data file.

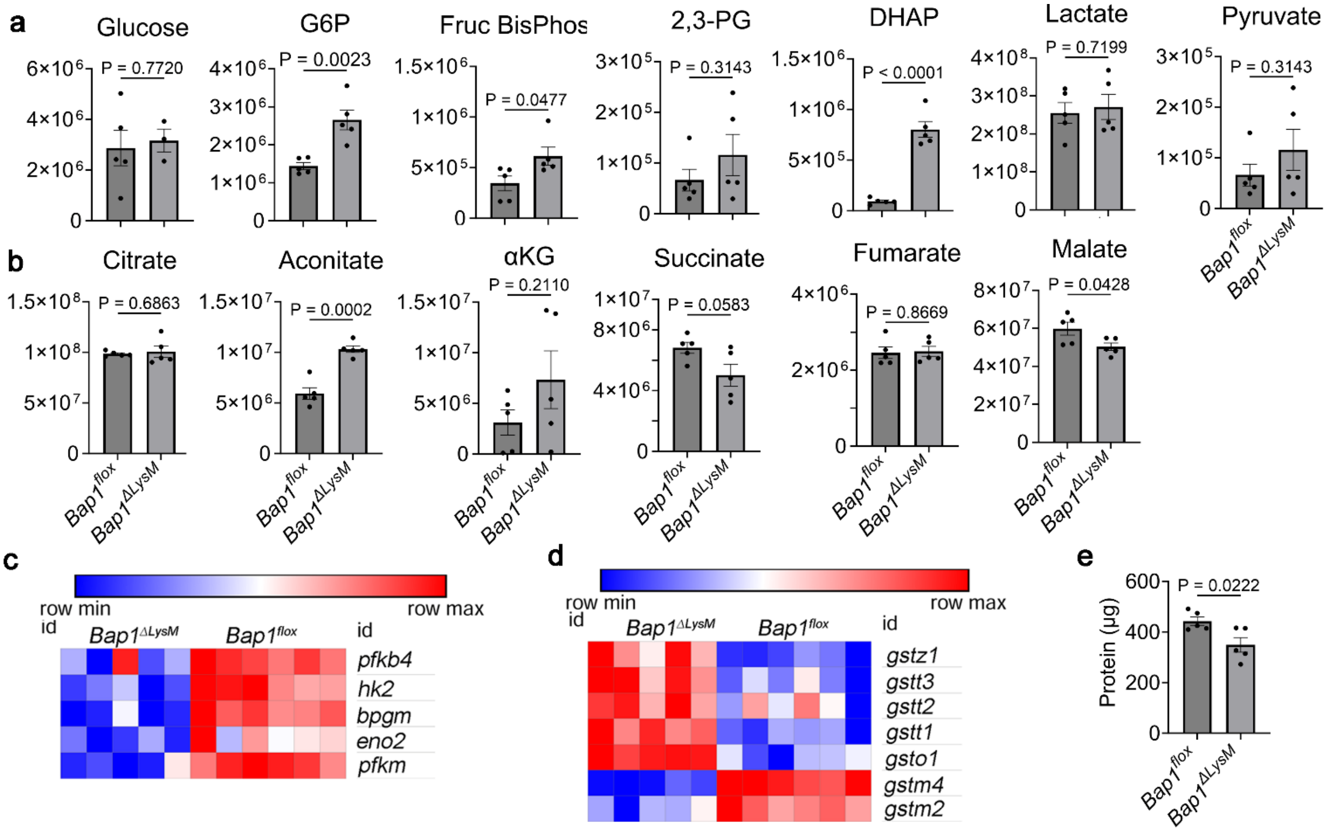

Supplementary Fig. 7: Bap1 regulates metabolic reprogramming in osteoclast. a Relative abundance of glutamine, aspartate and glutamate in the cell determined by LC-MS analysis. **b** heatmap of downregulated glycolytic genes. **c** heatmap of upregulated gst genes and **d** Protein content by genotype (n=5 biologically independent samples). Data represents mean $\pm$ SEM. P values shown are determined by two-sided Student *t* test (**a, d**). Source data are provided as a Source Data file.

Supplementary Table 1: Oligonucleotide List

**Primer name**	**type**	**5'-3'**
B4galt4 For	qPCR	AACCCACCTTATCACCTCTCC
B4galt4 Rev	qPCR	TAGCACCCACAAAGTAGTTGC
Bap1C91A For	SDM	CAGCTGATACCCAACTCTGCTGCAACTCATGCCTTG
Bap1C91A Rev	SDM	CAAGGCATGAGTTGCAGCAGAGTTGGGTATCAGCTG
bglobin For	qPCR-DNA	GAAGCGATTCTAGGGAGCAG
bglobin Rev	qPCR-DNA	GGAGCAGCAGCGATTCTGAGTAGA
CathepsinK For	qPCR	AGGCAGCTAAATGCAGAGGGTACA
CathepsinK Rev	qPCR	AGCTTGCATCGATGGACACAGAGA
Cox1 For	qPCR	TTTTCAGGCTTCACCCTAGATGA
Cox1 Rev	qPCR	GAAGAATGTTATGTTTACTCCTACGAA
Cox3 For	qPCR	CGGAAGTATTTTCTTTGCAGGAT
Cox3 Rev	qPCR	CAGCAGCCTCCTAGATCATGTG
CytB1 For	qPCR	GCCACCTTGACCCGATTCT
CytB1 Rev	qPCR	TTGCTAGGGCCGCGATAAT
DCStamp For	qPCR	ACTAGAGGAGAAGTCCTGGGAGTC
DCStamp Rev	qPCR	CACCCACATGTAGAGATAGGTCAG
Ehhadh For	qPCR	ATGGCTGAGTATCTGAGGCTG
Ehhadh Rev	qPCR	GGTCCAAACTAGCTTTCTGGAG
Ephx1 For	qPCR	GGAGACCTTACCACTTGAAGATG
Ephx1 Rev	qPCR	GCCCGGAACCTATCTATCCTCT
Fabp7 For	qPCR	GGACACAATGCACATTCAAGAAC
Fabp7 Rev	qPCR	CCGAACCACAGACTTACAGTTT
Gapdh For	qPCR	AGGTCGGTGTGAACGGATTTG
Gapdh Rev	qPCR	TGTAGACCATGTAGTTGAGGTCA
Gclc For	qPCR	GGGGTGACGAGGTGGAGTA
Gclc Rev	qPCR	GTTGGGGTTTGTCCTCTCCC
Ggt5 For	qPCR	TTCAATGGGACAGAAACCTTGAG
Ggt5 Rev	qPCR	TCCCTGTGTATAAGACCTCCG
Gss For	qPCR	CAAAGCAGGCCATAGACAGGG
Gss Rev	qPCR	AAAAGCGTGAATGGGGCATAC
Gsta3 For	qPCR	AAGAATGGAGCCTATCCGGTG
Gsta3 Rev	qPCR	CCATCACTTCGTAACCTTGCC
hBAP1 For	qPCR	GATACGTCCGTGATTGATGATGA
hBAP1 Rev	qPCR	TGAGTTGCACAAGAGTTGGGTA
integrinb3 For	qPCR	TTCGACTACGGCCAGATGATT
integrinb3 Rev	qPCR	GGAGAAAGACAGGTCCATCAAGT
mBap1 For	qPCR	CTCCTGGTGGAAGATTTCGGT
mBap1 Rev	qPCR	GAGTGGCACAAGAGTTGGGAA
mtCO2 For	qPCR-DNA	CCGACTAAATCAAGCAACA

**Primer Name**	**Type**	**5'-3'**
mtCO2 Rev	qPCR-DNA	CAATGGGCATAAAGCTATGG
Nd4 For	qPCR	CATCACTCTATTCTGCCTAGCAA
Nd4 Rev	qPCR	TCCTCGGGCCATGATTATAGTAC
Nfatc1 For	qPCR	CCCGTCACATTCTGGTCCAT
Nfatc1 Rev	qPCR	CAAGTAACCGTGTAGCTGCACAA
Pgc1b For	qPCR	CTCCAGGCAGGTTCAACCC
Pgc1b Rev	qPCR	GGGCCAGAAGTTCCCTTAGG
Siglec1 For	qPCR	CAGGGCATCCTCGACTGTC
Siglec1 Rev	qPCR	GGAGCATCGTGAAGTTGGTTG
Slc6a12 For	qPCR	GGTCCCTGAGGAAGGAGAGAT
Slc6a12 Rev	qPCR	GGGGATGAAGAAAGCTCCACC
Slc6a4 For	qPCR	TATCCAATGGGTACTCCGCAG
Slc6a4 Rev	qPCR	CCGTTCCCCTTGGTGAATCT
Slc6a5 for	qPCR	CCACCGGGATAGTCCTCGT
Slc6a5 Rev	qPCR	GGCTGCTGAGATTACAAAACCC
Slc7a11 For	qPCR	GGCACCGTCATCGGATCAG
Slc7a11 Rev	qPCR	CTCCACAGGCAGACCAGAAAA
Slc7a11 promoter 2 For	ChIP qPCR	CCAAGACTTTCACCTGGAAAGGATA
Slc7a11 promoter 2 Rev	ChIP qPCR	GAGACTGCACAGGGAATACAC
Slc7a11 promoter distant For	ChIP qPCR	TGAAGACACGCACAGAGCAA
Slc7a11 promoter distant Rev	ChIP qPCR	ATTTCCGGCTTTGAGCTAAC
Slc7a11 promoter near For	ChIP qPCR	CGTGGAAGGCTCCGTATTTA
Slc7a11 promoter near Rev	ChIP qPCR	TAATGTTGGCGCTTTCTCAA

98

Supplementary Table 2: Antibody List

Antibody	Company	Catalog No.	Lot No.	Dilution	Application	Validation
Alexa 488 Phalloidin	Thermo Scientific	A12379	1360987	1:60	IFC	Mouse
Actin	Sigma Aldrich	A2228	476697	1:10,000	WB	Mouse
Bap1	Cell Signaling Technologies	13187s	1	1:1000<br>5ug	WB<br>ChIP	Human
CathepsinK	EMD Millipore	MAB3324 (182-12G5)	2519359	1:1000	WB	Human
H2AK119ub1	Cell Signaling Technologies	8240s(D27C4xp)	8	1:2000<br>5ug	WB<br>ChIP	Mouse
Normal Mouse IgG	SantaCruz Biotechnologies	Sc2025	LH0615	5ug	ChIP	Mouse
Normal Rabbit IgG	SantaCruz Biotechnologies	Sc2027	B0515	5ug	ChIP	Mouse
Integrinb3	Cell Signaling Technologies	4702s	5	1:1000	WB	Mouse
NFATc1	SantaCruz Biotechnologies	Sc7294(7A6)	12214	1:100	WB	Mouse
pSrc	Cell Signaling Technologies	2010L	17	1:1000	WB	Mouse
Rac1	Thermo Scientific	1862341	we328014	1:1000	IP/WB	Mouse
ubiquitin	Cell Signaling Technologies	3936s (P4D1)	18	5ug	IP	Mouse
VDAC	Cell Signaling Technologies	4661s (D73D12)	10	1:1000	WB	Mouse
HA tag	Covance	16B12		1:1000	WB	Mouse
Goat anti-Rabbit IgG Alexa Fluor™ 680	Thermo Scientific	21109	37505A	1:5000	WB	Rabbit
Goat anti-Mouse IgG Alexa Fluor™ 680	Thermo Scientific	21058	35818A	1:5000	WB	Mouse
Rabbit IgG DyLight™ 800 Donkey Polyclonal	Rockland	611-732-127	25342	1:5000	WB	Rabbit
Mouse IgG DyLight™ 800 Donkey Polyclonal	Rockland	611-432-042	18247	1:5000	WB	Mouse

Uncropped Immunoblots used in supplementary figures:

Extended Data **3b** pSrc (Y416) and Actin blot

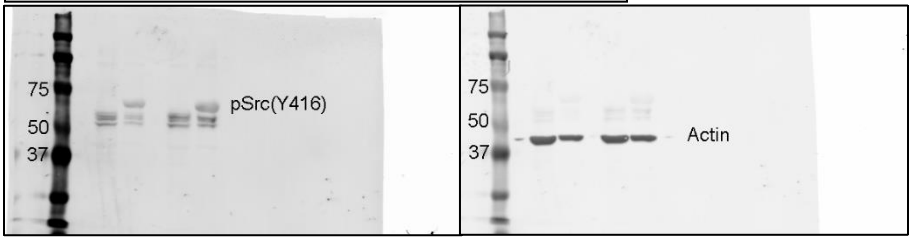

Extended Data **3c** VDAC and Actin blot

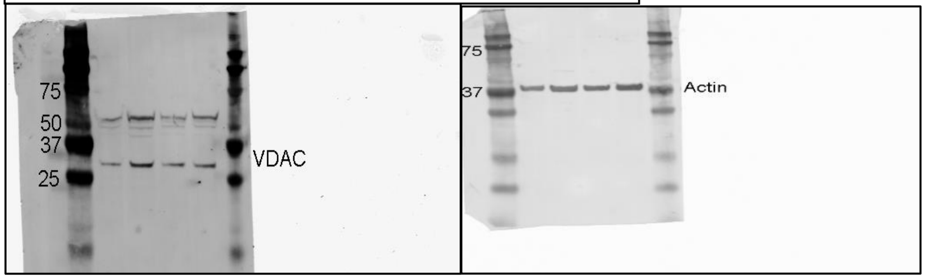

Extended Data **5c**: SLC7A11 and Actin

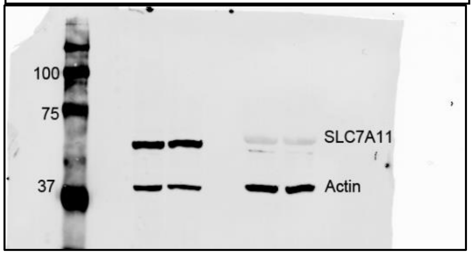

Extended Data **6d**:HA-PGC1b and Actin

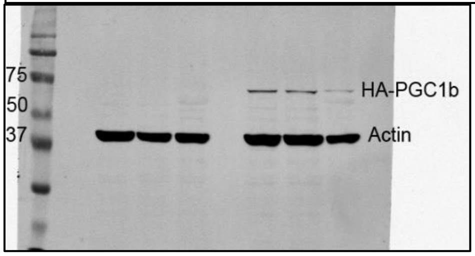

Supplement: Supplementary file 1 — Supplementary Information [file 41467_2023_41629_MOESM1_ESM.pdf]
