# Supplementary material for: BAP1 promotes osteoclast function by metabolic reprogramming
Source: Nat Commun. 2023 Sep 22;14:5923. doi: 10.1038/s41467-023-41629-4 (PMC10516877; doi:10.1038/s41467-023-41629-4)
Supplement: Supplementary file 2 — Description of Additional Supplementary Files Document [file 41467_2023_41629_MOESM2_ESM.pdf]

## Description of Additional Supplementary Files

**Supplementary Data1:** Metabolomics data from Bap1flox and Bap1ΔLysM osteoclasts as determined by LC-MS analysis; n=5 biologically independent samples from 20-week-old male mice of either genotype.
