# Supplementary material for: BAP1 promotes osteoclast function by metabolic reprogramming
Source: Nat Commun. 2023 Sep 22;14:5923. doi: 10.1038/s41467-023-41629-4 (PMC10516877; doi:10.1038/s41467-023-41629-4)
Supplement: Supplementary file 4 — Reporting Summary [file 41467_2023_41629_MOESM4_ESM.pdf]

nature portfolio

Corresponding author(s): Nidhi Rohatgi

Last updated by author(s): Aug 30, 2023

Reporting Summary

Nature Portfolio wishes to improve the reproducibility of the work that we publish. This form provides structure for consistency and transparency in reporting. For further information on Nature Portfolio policies, see our Editorial Policies and the Editorial Policy Checklist.

Statistics

For all statistical analyses, confirm that the following items are present in the figure legend, table legend, main text, or Methods section.

n/a	Confirmed	
☐	☒	The exact sample size (*n*) for each experimental group/condition, given as a discrete number and unit of measurement
☐	☒	A statement on whether measurements were taken from distinct samples or whether the same sample was measured repeatedly
☐	☒	The statistical test(s) used AND whether they are one- or two-sided<br>*Only common tests should be described solely by name; describe more complex techniques in the Methods section.*
☐	☒	A description of all covariates tested
☐	☒	A description of any assumptions or corrections, such as tests of normality and adjustment for multiple comparisons
☐	☒	A full description of the statistical parameters including central tendency (e.g. means) or other basic estimates (e.g. regression coefficient) AND variation (e.g. standard deviation) or associated estimates of uncertainty (e.g. confidence intervals)
☐	☒	For null hypothesis testing, the test statistic (e.g. *F*, *t*, *r*) with confidence intervals, effect sizes, degrees of freedom and *P* value noted<br>*Give P values as exact values whenever suitable.*
☒	☐	For Bayesian analysis, information on the choice of priors and Markov chain Monte Carlo settings
☒	☐	For hierarchical and complex designs, identification of the appropriate level for tests and full reporting of outcomes
☐	☒	Estimates of effect sizes (e.g. Cohen's *d*, Pearson's *r*), indicating how they were calculated

Our web collection on statistics for biologists contains articles on many of the points above.

Software and code

Policy information about availability of computer code

Data collection	MetaboAnalyst 5.0,<br>Image J version 1.53k,<br>Bioquant osteo 2021,<br>Nanozoomer (model 2.0HT) for image acqisition<br>Nikon DS-U2/L2 USB version 5.547<br>R/Bioconductor Package version 3.17<br>Skyline version 22.2<br>Wave version 2.6.1.53
Data analysis	Fiji Image processing package for viewing and scoring; Graphpad Prism version 10.0 for statistical analysis.

For manuscripts utilizing custom algorithms or software that are central to the research but not yet described in published literature, software must be made available to editors and reviewers. We strongly encourage code deposition in a community repository (e.g. GitHub). See the Nature Portfolio guidelines for submitting code & software for further information.

Data

Policy information about availability of data

All manuscripts must include a data availability statement. This statement should provide the following information, where applicable:

- Accession codes, unique identifiers, or web links for publicly available datasets
- A description of any restrictions on data availability
- For clinical datasets or third party data, please ensure that the statement adheres to our policy

The RNA-seq data generated in this study have been deposited in the Gene Expression Omnibus database under the accession code "https://www.ncbi.nlm.nih.gov/geo/query/acc.cgi?acc=GSE231819". The metabolomic data in this study are provided as a supplementary data file. Source data are provided as a Source Data file.

Research involving human participants, their data, or biological material

Policy information about studies with human participants or human data. See also policy information about sex, gender (identity/presentation), and sexual orientation and race, ethnicity and racism.

Reporting on sex and gender	N/A
Reporting on race, ethnicity, or other socially relevant groupings	N/A
Population characteristics	N/A
Recruitment	N/A
Ethics oversight	N/A

Note that full information on the approval of the study protocol must also be provided in the manuscript.

Field-specific reporting

Please select the one below that is the best fit for your research. If you are not sure, read the appropriate sections before making your selection.

☒ Life sciences ☐ Behavioural & social sciences ☐ Ecological, evolutionary & environmental sciences

For a reference copy of the document with all sections, see nature.com/documents/nr-reporting-summary-flat.pdf

Life sciences study design

All studies must disclose on these points even when the disclosure is negative.

Sample size	For invivo studies, based on our preliminary data in similar approaches, we studied 5-13 animals in most experiments/ group to achieve 80% power with a 2-tailed P value of 0.05. For invitro data, sample size calculation was not performed. Sizes we chose throughout the manuscript were based on preliminary experiments and were considered sufficient as long as differences or lack of differences were statistically significant and reproducible in independent experiments. All n values are indicated in figure legends.
Data exclusions	There were no data exclusions
Replication	To assure scientific rigor and reproducible unbiased data, each analysis of cells or tissues was performed in triplicates and repeated atleast two-three times with similar results.
Randomization	Samples and cell culture experiments used for control and experiment were allocated randomly.
Blinding	The invivo analysis was conducted by a blinded researcher. Invitro experiments were not blinded as data analysis is based off objectively measureable data such as TRAP staining, presence or absence of actin rings, protein expression or metabolic changes. RNAseq and metabolomic analysis was conducted by a blinded researcher.

Reporting for specific materials, systems and methods

We require information from authors about some types of materials, experimental systems and methods used in many studies. Here, indicate whether each material, system or method listed is relevant to your study. If you are not sure if a list item applies to your research, read the appropriate section before selecting a response.

Materials & experimental systems

n/a	Involved in the study	
☐	☒	Antibodies
☐	☒	Eukaryotic cell lines
☒	☐	Palaeontology and archaeology
☐	☒	Animals and other organisms
☒	☐	Clinical data
☒	☐	Dual use research of concern
☒	☐	Plants

Methods

n/a	Involved in the study	
☒	☐	ChIP-seq
☒	☐	Flow cytometry
☒	☐	MRI-based neuroimaging

Antibodies

Antibodies used

anti-Bap1(Cell Signaling Technologies, Cat No. 13187s, Lot No. 1); anti-Integrinb3(Cell Signaling Technologies, Cat No.4702s, Lot No.5); anti-NFATc1(SantaCruz Biotechnologies, CatNo. sc7294(7A6), Lot No.12214); anti-H2Ak119ub1(Cell Signaling Technologies, Cat No. 8240s(D27C4xp), Lot No.8); anti-CathepsinK (EMD Millipore, Cat No. MAB3324 (182-12G5), Lot No.2519359); Alexa 488 (Thermo Scientific, Cat No. A12379, Lot No.1360987); anti-Rac1 (Thermo Scientific, Cat No. 1862341, Lot No.we328014); anti-pSrc (Cell Signaling Technologies, Cat No. 2010L, Lot No. 17); anti-Actin (Sigma Aldrich, CatNo. A2228, Lot #476697); anti-IgG Mouse(SantaCruz Biotechnologies, CatNo. Sc2025, Lot No.H0615); anti-IgG Rabbit(SantaCruz Biotechnologies, CatNo. Sc2027, Lot No.B0515); anti-ubiquitin (Cell Signaling Technologies, Cat No. 3936s (P4D1), Lot No. 18), anti-VDAC (Cell Signaling Technologies, Cat No. 4661s (D73D12), Lot No.10); Goat anti-Rabbit IgG Alexa Fluor™ 680 (Thermo Scientific, Cat No. 21109, Lot No. 37505A); Goat anti-Mouse IgG Alexa Fluor™ 680 (Thermo Scientific, Cat No. 21058, Lot No. 35818A); Rabbit IgG DyLight™ 800 Donkey Polyclonal (Rockland, Cat No. 611-732-127, Lot No. 25342); Mouse IgG DyLight™ 800 Donkey Polyclonal (Rockland, Cat No. 611-432-042, Lot No. 18247).

Validation

All antibodies that were used in the manuscript have been validated for use in mouse tissues on supplier website and for immunoblot and IP applications. BAP1 antibody has been validated to cross react to human Bap1 constructs on the manufacturer website. This antibody was only used to detect overexpressed human BAP1 in our invitro model.

Eukaryotic cell lines

Policy information about cell lines and Sex and Gender in Research

Cell line source(s)

293 T cells (ATCC, Cat No. CRL3216); Platinum E cells (Cell Biolabs, Cat No. RV101)

Authentication

None of the cell lines used were authenticated

Mycoplasma contamination

Cell lines were not tested for mycoplasma contamination

Commonly misidentified lines
(See ICLAC register)

We have not used any commonly misidentified cell lines listed in the ICLAC Table

Animals and other research organisms

Policy information about studies involving animals; ARRIVE guidelines recommended for reporting animal research, and Sex and Gender in Research

Laboratory animals

All animals were housed in the animal care unit of Washington University School of Medicine, where they were maintained at 22C in a 12-hour light-dark cycle according to guidelines of the Association for Assessment and Accreditation of Laboratory Animal Care. All mice used for experiments were healthy and had free access to water and food. All mice were from in house matings. Animal work was performed according to the policies of Animal Studies Committee (ASC) at Washington University School of Medicine in St. Louis. Mice were analyzed under approved protocols (Protocol No. 23-0120) and were provided appropriate care while undergoing research which complies with the standards in the Guide for the Use and Care of Laboratory Animals and the Animal Welfare Act.

Bap1flox mice were kindly provided by Prof. Anwesha Dey, Genentech Inc. Bap1ΔLysM (deletion in all myeloid cells) or Bap1ΔcatKmice (deletion in mature osteoclasts) mice were developed by breeding homozygous for floxed Bap1 with mice expressing Cre recombinase under the influence of LysozymeM Cre promoter or CathepsinK promoter as previously reported. The age of mice for most experiments was 8-20 week, but for those studies requiring mice to be 1 year old. Both males and females were evaluated.

Wild animals

No wild animals were used in the study.

Reporting on sex

Invivo studies were conducted in both males and females separately. No gender differences were noted.

Field-collected samples

Study did not involve samples collected from the field

Ethics oversight

Mice were housed in the animal care unit of Washington University School of Medicine, where they were maintained according to

Ethics oversight	guidelines of the Association for Assessment and Accreditation of Laboratory Animal Care. All animal experimentation was approved by the Animal Studies Committee of Washington University School of Medicine.

Note that full information on the approval of the study protocol must also be provided in the manuscript.
